# LC-MS-based metabolomics for detecting adulteration in *Tribulus terrestris*-derived dietary supplements^☆^

**DOI:** 10.1016/j.fochx.2025.102476

**Published:** 2025-04-18

**Authors:** Dejan Gođevac, Jovana Stanković Jeremić, Mirjana Cvetković, Katarina Simić, Ivana Sofrenić, Jovana Ljujić, Lazar Popović, Uroš Gašić, Yen-Nhi Hoang, Tao Huan, Stefan Ivanović

**Affiliations:** a University of Belgrade, Institute of Chemistry, Technology and Metallurgy – National Institute of the Republic of Serbia, Njegoševa 12, 11000 Belgrade, Serbia; b University of Belgrade – Faculty of Chemistry, Studentski trg 12-16, 11000 Belgrade, Serbia; c University of Belgrade – Innovation Center of Faculty of Chemistry, Studentski trg 16, 11000 Belgrade, Serbia; d University of Belgrade – Institute for Biological Research “Siniša Stanković” – National Institute of Republic of Serbia, Bulevar Despota Stefana 142, 11000 Belgrade, Serbia; e Department of Chemistry, Faculty of Science, University of British Columbia, Vancouver Campus, 2036 Main Mall, Vancouver, BC V6T 1Z1, Canada

**Keywords:** *Tribulus terrestris*, Metabolomics, LC-HRMS, Dietary supplements, Adulteration

## Abstract

The widespread usage of *Tribulus terrestris* dietary supplements has elicited concerns over product authenticity and possible adulteration. This research utilized an untargeted liquid chromatography-high resolution mass spectrometry (LC-HRMS) metabolomics methodology to assess the composition of *T. terrestris*-derived supplements. Authentic plant materials, simulated adulterated samples, and commercial products were analyzed using principal component analysis (PCA), orthogonal partial least squares discriminant analysis (OPLS-DA), and a convolutional neural network tool. The presence of PDE5 inhibitors and anabolic steroids in spiked samples was confirmed. Certain commercial products included undisclosed green tea and citrus-derived chemicals, likely incorporated to amplify stimulant effects and support testosterone-enhancing claims. Additionally, spirost-4-ene-3,12-dione was recognized as an indicator of possible steroidal saponin decomposition resulting from storage or processing conditions. This study illustrates the efficacy of LC-HRMS metabolomics in detecting supplement adulteration and emphasizes the need for rigorous quality control protocols to guarantee customer safety and product integrity.

## Highlights

• LC-HRMS metabolomics detects adulterants in *Tribulus terrestris* supplements.
• PDE5 inhibitors and anabolic steroids were confirmed in spiked samples.
• Undisclosed green tea and citrus compounds found in commercial products.
• Spirost-4-ene-3,12-dione suggests steroidal saponin degradation.
• Findings highlight the need for more rigorous supplement quality control.

## 1 Introduction

*Tribulus terrestris* L. is a creeping, herbaceous annual plant from the Zygophyllaceae family, native to warm climates across Europe, Asia, Africa, America, and Australia. *T. terrestris* is an important medicinal plant with a long history of use in traditional medicine worldwide (Ghosh et al., 2012; Zahedi et al., 2024). It has been used to treat eye infections (Ghosh et al., 2012), abdominal bloating (Zahedi et al., 2024), swelling, pathological pains, edema (Ghosh et al., 2012), kidney disorders (Ghosh et al., 2012; Zahedi et al., 2024), cardiovascular system diseases (Zahedi et al., 2024), gastrointestinal liver diseases (Zahedi et al., 2024), itchy skin (Saeed et al., 2024), urinary tract infections, heart issues, and high blood pressure (Saeed et al., 2024). *T. terrestris* extracts have exhibited a range of pharmacological effects, including hypercholesteremic, antioxidant, antibacterial, anti-inflammatory, analgesic, and hepatoprotective properties (Saeed et al., 2024), free radical scavenging activity (Zahedi et al., 2024), as well as antihypertensive and antifungal effects (Ghosh et al., 2012; Zahedi et al., 2024). Additionally, the extracts demonstrated diuretic, anthelmintic, and anticancer effects (Ghosh et al., 2012; Saeed et al., 2024), provided protective effects against ischemic stroke, and demonstrated antispasmodic and immunomodulatory properties (Saeed et al., 2024). Globally, extracts of *T. terrestris* have been primarily used to enhance muscle strength and to treat impotence and sexual dysfunction (Saeed et al., 2024; Zahedi et al., 2024). The wide spectrum of biological activity of *T. terrestris* extracts comes from their rich and varied chemical composition, which includes steroidal saponins, flavonoids, alkaloids, tannins, and phenolic acids (Ghosh et al., 2012; Saeed et al., 2024; Zahedi et al., 2024).

The popularity of dietary supplements has increased dramatically in the last several years. Herbal supplements are particularly appealing to consumers because they are marketed as a natural alternative to treat nutrient deficiencies (Taghvimi et al., 2019), perceived as safer and healthier than synthetic drugs, and commonly used for weight loss, muscle building, and boosting energy levels (Taghvimi et al., 2019). *T. terrestris* dietary supplementation effectively reduces inflammation and oxidative stress, improves muscle tone, and supports sexual function in men (Saeed et al., 2024).

Despite strict quality control regulations on the production and labeling of herbal supplements, the counterfeiting of botanical dietary supplements remains a widespread problem (Walker & Applequist, 2012). These preparations frequently become the focus of fraud, primarily driven by economic motivations (Jiru et al., 2019). However, some manufacturers intentionally or negligently adulterate these products unlawfully, leading to serious or even tragic health consequences. As a result, the effectiveness and safety of these supplements are increasingly being questioned (Walker & Applequist, 2012; Kioukia-Fougia et al., 2016; Stefanescu et al., 2020). The addition of synthetic drugs or their analogs into naturally sourced products to ensure or augment specific pharmacological effects is a common form of adulteration (Jiru et al., 2019). This leads to certain commercially accessible food supplements experiencing significant quality issues, including misleading advertising of their benefits and inaccurate information regarding the functional ingredients (Chen et al., 2023).

The identification and characterization of adulterants in food supplements have been achieved using various analytical techniques, particularly genetic methods, nuclear magnetic resonance (NMR), and liquid chromatography-mass spectrometry (LC-MS). Spectroscopic methods, including Fourier transform infrared (FTIR) spectroscopy – using techniques such as attenuated total reflection and differential spectroscopy – together with hyperspectral imaging (HSI), have been employed in the detection of food adulteration (Xie et al., 2021).

Genetic tests, such as digital droplet PCR (ddPCR), real-time PCR (qPCR), polymerase chain reaction restriction fragment length polymorphism (PCR-RFLP), and multiplex PCR are valuable techniques for both qualitative and quantitative analysis of adulterants (Jiang et al., 2024). However, these tests are confined to adulterants that include DNA (such as contaminants from plants, animals, or microbes) and they cannot detect non-biologically produced substances like synthetic chemicals, heavy metals, or specific medications. Additionally, an unknown or novel adulterant cannot be identified without prior knowledge of its genetic material (Gloyn et al., 2012). NMR techniques offer a more practical, accurate, and precise analysis with simpler sample preparation compared to other methods. However, its requirement for highly skilled professionals, high cost, and low sensitivity can limit its widespread adoption (Chen et al., 2023; Pujol et al., 2024). FTIR spectroscopy offers a fast, non-destructive method for studying molecular structures; however, its utility is often limited by overlapping spectrum characteristics and difficulties in identifying trace-level adulterants (Sota-Uba et al., 2021). HSI is a robust analytical method that combines traditional imaging and spectroscopy, providing both spatial and spectral data from an object. Recent advancements such as multi-molecular IR (MM-IR) spectroscopy enable high-throughput detection of multiple trace adulterants. By integrating hyperspectral imaging with spectral enhancement techniques, MM-IR improves qualitative and quantitative detection. However, it struggles with low-concentration adulterants in complex herbal supplements (Xie et al., 2021). LC-MS, particularly high-resolution mass spectrometry (HRMS), has emerged as a powerful tool for detecting adulterants due to its high sensitivity, specificity, and ability to provide detailed chemical profiles (Roiffé et al., 2019; Lv et al., 2024; Jiang et al., 2024; Wallace et al., 2020). A more comprehensive, holistic approach to analyzing adulterants in supplements is based on a metabolomics approach, which combines chemometrics with the results of the described methods (Roiffé et al., 2019). The integration of spectroscopic or spectrometric and chromatographic data with multivariate statistical methods has reduced complexity, facilitating a wide range of applications for detecting adulteration in complex mixtures (Hosseini et al., 2022; Trbović et al., 2017).

Our research introduces an advanced untargeted metabolomics approach employing Orbitrap HRMS for the rapid and comprehensive identification of adulterants in *T. terrestris*-based dietary supplements. This technology, in contrast to traditional approaches that typically focus on targeted screening of known contaminants, allows for the detection of both expected and unexpected adulterants with exceptional sensitivity and specificity. To establish and verify this methodology, three categories of samples were examined: (i) authentic plant material, (ii) simulated fraudulent products where authentic plant material was blended with known adulterants to mimic adulteration, and (iii) commercially available supplements of unknown authenticity.

This method utilizes multivariate statistical techniques, including principal component analysis (PCA) and partial least squares (PLS), to efficiently distinguish between authentic and adulterated samples based on their metabolic profiles. Additionally, a convolutional neural network (CNN)-based filtering tool (Xing et al., 2021) was used to improve the detection of steroid-related compounds, given that steroidal saponins are the principal bioactive components of *T. terrestris*, while anabolic steroids are a common form of its adulteration. This workflow enhances quality control initiatives and offers a more efficient and high-throughput approach to ensure consumer safety and regulatory compliance in the dietary supplement sector.

## 2 Materials and methods

### 2.1 Chemicals

Ethanol, methanol, and acetonitrile of HPLC grade were purchased from Sigma Aldrich. Germany. Ultrapure water (18.2 MΩcm^−1^) was obtained from Arium Mini Ultrapure Water System (Sartorius, Germany). Sildenafil citrate, tadalafil, vardenafil hydrochloride, testosterone propionate, and 4-androstene-3,17-dione, all with a purity of ≥98 %, were purchased from Sigma Aldrich, Germany.

### 2.2 Sample collection

Plant samples of *T. terrestris* were collected from 11 locations in Serbia, Greece, and Bulgaria, and three dry plant samples were obtained from a pharmaceutical company in Turkmenistan. Multiple biological replicates were collected from various sites, yielding a total of 38 samples consisting of fruits or entire aerial parts. Voucher specimens have been deposited at the Institute for Medicinal Plants Research “Dr. Josif Pančić”, Belgrade, Serbia. The details are provided in Table S1.

Additionally, 27 commercially available supplements, reportedly containing *T. terrestris* extract in various pharmaceutical forms, were obtained for analysis. Of these, 23 samples were manufactured in Europe, two in the UK, and two in the USA. The declared content of steroid saponins ranged from 20 to 95 % (Table S2).

### 2.3 Sample preparation

Plant samples were first air-dried and then finely ground using an analytical mill (IKA A11 basic, Germany). For extraction, 2 g of aerial part samples, 1 g of fruit samples, and 2 g of supplement samples were utilized. The extraction was performed with 15 mL of 70 % EtOH in an ultrasonic bath (Elmasonic P 30H, Germany) for 30 min at 80 °C. The ethanolic extracts were filtered and evaporated under reduced pressure. The resulting extracts were further purified using solid-phase extraction (SPE) with 500 mg/6 mL C18-E cartridges (Strata, Phenomenex, USA). Conditioning was done with 5 mL of methanol, followed by equilibration with 5 mL of water. Subsequently, the dissolved extracts (20 mg in 10 mL of water) were loaded into the cartridges, washed with 5 mL of 5 % methanol in water, and analytes were eluted with 5 mL of methanol. The final extracts were filtered through a 0.45-μm nylon filter (Agilent, US) before LC-HRMS analysis.

Two stock solutions were prepared to simulate adulteration. The first contained a mixture of phosphodiesterase-5 inhibitors – sildenafil, tadalafil, and vardenafil – each at a concentration of 5 % in methanol (PDE5i mix). The second consisted of anabolic steroids, specifically testosterone propionate, and 4-androstene-3,17-dione, each at a concentration of 5 % in methanol (steroid mix). Seven randomly selected pulverized plant samples (2 g of aerial parts and 1 g of fruit) were individually spiked with 100 μL of the PDE5i mix, while another seven were spiked with 100 μL of the steroid mix to simulate adulteration. Extraction and SPE were then performed as previously described.

### 2.4 LC-HRMS analysis

In total, 79 samples were prepared for LC-HRMS analysis, 38 authentic, 14 spiked samples, and 27 commercially available supplements. A QC solution was prepared by combining 100 μL of each tested sample, ensuring consistent and reliable comparison of results across all runs. The PCA score plot (Fig. S11) illustrates that all five QC sample runs are tightly clustered near the origin, indicating high reproducibility of the LC-HRMS analysis.

The LC-HRMS analysis was conducted using a Thermo Scientific™ Vanquish™ Core HPLC system coupled to the Orbitrap Exploris 120 mass spectrometer (San Jose, CA, USA).

The elution was performed at 25 °C on a Hypersil GOLD™ C18 analytical column (50 × 2.1 mm, 1.9 mm) from Thermo Fisher Scientific. The mobile phase consisted of (A) a 0.1 % aqueous formic acid solution and (B) acetonitrile MS grade containing 0.1 % formic acid, which were applied in the following gradient program: 5 % B in the first 1.12 min, 5–20 % B from 1.12 to 1.68 min, 20–80 % B from 1.68 to 7.26 min, 80–95 % B from 7.26 to 7.82 min, 95 % B from 7.82 to 9.94 min, 95 %–5 % B from 9.94 to 10.00 min, and 5 % B until the 13th min. The flow rate was set to 0.4 mL/min and the injection volume was 3 μL.

The Orbitrap Exploris 120 mass spectrometer was equipped with an ESI source operating in positive ionization mode. The capillary voltage, nebulizer gas pressure, drying gas flow rate, and source temperature were described by Xing et al. (2021). Full scan MS was monitored from 100 to 1500 *m*/*z* with the Orbitrap resolution set to 60,000 FWHM, RF Lens 70 %, and maximum injection time 100 ms, while data-dependent MS2 experiments were monitored from 50 m/z with an Orbitrap resolution of 15,000 FWHM and normalized collision energy set to 30 %, isolation window 1.5 m/z, and maximum injection time 22 ms. The dynamic exclusion time was set to 5 s, with exclusion applied after one occurrence of a specific scan. The intensity threshold was set to 5 × 10^3^.

Data was acquired using the Xcalibur® data software (Thermo Finnigan, San Jose, CA, USA).

### 2.5 Data preprocessing and multivariate data analysis

MS DIAL software version 5.5 (Tsugawa et al., 2015) was used for feature extraction and alignment. Mass tolerances of 0.01 and 0.025 Da for MS1 and MS2, respectively, were used during spectral centroiding. For peak detection, a minimum peak height of 3,000,000 and a mass slice width of 0.1 Da were applied. Peaks were then aligned across samples using an MS1 tolerance of 0.015 Da and a retention time tolerance of 0.1 min, implementing a 5-fold sample average/blank average change filter. Total ion current (TIC) normalization to account for variations in sample concentration and instrument response was applied and the alignment results were exported in .csv format for subsequent multivariate data analysis. Compound annotation was performed using MS-FINDER software (Tsugawa et al., 2016).

To refine the dataset and identify steroid-like features, the SteroidXtract tool, a convolutional neural network (CNN)-based approach, was applied (Xing et al., 2021). Raw LC-HRMS data were converted to mzXML format using ProteoWizard msconvert software. SteroidXtract took mzXML files as the input and generated prediction scores for each MS2 feature, with scores above 0.5 indicating steroid-like features. A Python script was then used to integrate positive SteroidXtract predictions with MS-DIAL data based on retention time and precursor *m*/*z* tolerances. The updated feature table, annotated with prediction scores and the sample(s) from which each feature was detected, was used for further analysis.

SIMCA software (version 17, Sartorius Stedim Biotech Goettingen, Germany) was used for multivariate data analysis. The normalized LC-HRMS data were mean-centered, without scaling.

## 3 Results and discussion

### 3.1 LC-HRMS metabolomics profiling

To develop an untargeted metabolomics approach for detecting adulterants in *T. terrestris*-based dietary supplements, three categories of samples were obtained: i) authentic plant material, ii) simulated fraudulent plant material, and iii) commercially available supplements. To account for variations in metabolite composition due to ecological factors and differences in tissue composition, several biological replicates from different botanical populations were used in the selection of authentic plant material. Therefore, *T. terrestris* specimens were gathered from distinct places, across various years and months, and from diverse plant parts, including either fruits or the whole aerial parts (Table S1).

In recent years, numerous reports have documented the adulteration of aphrodisiac dietary supplements, primarily with active pharmaceutical ingredients from phosphodiesterase-5 (PDE5) inhibitors, such as sildenafil, vardenafil, and tadalafil – compounds intended for the treatment of erectile dysfunction (Jiru et al., 2019; Pujol et al., 2024). To mimic such adulteration, these PDE5 inhibitors were selected for blending with authentic plant material. Given that *T. terrestris* extracts are also marketed for muscle-strengthening purposes, adulteration with anabolic agents – known for reducing body fat and increasing muscle mass – poses a plausible concern (Roiffé et al., 2019). Therefore, testosterone propionate and 4-androstene-3,17-dione were incorporated into authentic plant material to simulate adulteration.

Using an Orbitrap LC-HRMS-based untargeted methodology, all authentic and spiked samples, as well as commercially available supplements, were examined. A data-dependent acquisition (DDA) was used to preferentially fragment the most intense ions, generating high-quality MS/MS spectra to enhance metabolite identification (Guo et al., 2022). This methodology was appropriate, as the objective was not trace-level detection but rather the identification of adulteration involving significant or therapeutically relevant quantities of added adulterants.

Feature extraction and peak alignment in the positive mode LC-HRMS data were performed utilizing techniques integrated into MS-DIAL, an open-source LC-MS data processing software. To minimize false-positive metabolite feature detection, the extraction sensitivity was reduced by setting a high minimum peak height threshold. Furthermore, all extracted ion chromatograms were visually examined to confirm Gaussian-like peak shapes, thereby guaranteeing the reliability of the features found. Additionally, a five-fold sample average-to-blank average change filter was applied to further reduce false-positive detections. This resulted in a total of 1531 extracted features.

Compound annotation was performed using *in silico* fragmentation of all predicted molecular formulas, determined based on accurate mass, isotope ratios, and ion data obtained from databases integrated into the MS-FINDER software (Tsugawa et al., 2016). In cases where an equivalent feature in the negative MS mode was present, *in silico* analysis was conducted accordingly. The MS data of structural candidates generated by MS-FINDER were compared to those from the literature. Moreover, the botanical origin of the identified compounds was also verified through the literature. This approach enabled compound identification at confidence level 2, as outlined by the Compound Identification Work Group of the Metabolomics Society (Blaženović et al., 2018). The most important compounds identified in this study are listed in Table 1. The mass ppm error and MS/MS fragments for the adulterants identified are listed in Table S3.

**Table 1:** The main LC-MS data of the most important compounds identified.

Average Rt(min)	Average Mz pos. Mode	Adduct type pos. Mode	Adduct type neg. Mode	Molecular formula	Compound name	Compound ontology	Origin	Literature
3699	307.08154	[M + H]+	[M-H]-	C15H14O7	Epigallocatechin	Epigallocatechins	Green tea	Morikawa et al., 2013
3797	195.08765	[M + H]+	n.d.	C8H10N4O2	Caffeine	Xanthine alkaloids - CNS Stimulant	Green tea	Morikawa et al., 2013
3984	307.17651	[M + H]+	[M-H]-	C12H18O7S	12-Hydroxyjasmonic acid sulfate	Jasmonic acids	*T. terrestris*	Uysal et al., 2023
4002	227.12791	[M + H]+	[M-H]-	C12H18O4	12-Hydroxyjasmonic acid	Jasmonic acids	*T. terrestris*	Uysal et al., 2023
4.061	291.0867	[M + H]+	n.d.	C15H14O6	Epicatechin	Catechins	Green tea	Morikawa et al., 2013
4.366	611.15997	[M + H]+	[M-H]-	C27H30O16	Rutin	Flavonoid-3-O-glycosides	*T. terrestris*	Wang et al., 2024
4531	1079.5264	[M + H-H2O]+	[M-H]-	C51H84O25	Terrestrosin I	Steroidal saponins	*T. terrestris*	Wang et al., 2026
4675	581.18585	[M + H]+	[M-H]-	C27H32O14	Naringin	Flavonoid-7-O-glycosides	Citrus fruits	Elhady et al., 2024
4728	300.12262	[M + H]+	[M-H]-	C17H17NO4	N-trans-Caffeoyltyramine	Hydroxycinnamic acids	*T. terrestris*	Uysal et al., 2023
4746	435.12817	[M + H]+	[M-H]-	C21H22O10	Prunin	Flavonoid-7-O-glycosides	Citrus fruits	Elhady et al., 2024
4795	919.48865	[M + H-H2O]+	n.d.	C45H76O20	Terrestrosin F	Steroidal saponins	*T. terrestris*	Wang et al., 2023
4841	245.11743	[M + 2H]2+	n.d.	C23H32N6O4S	Vardenafil	Phosphodiesterase-5 inhibitors	Analytical standard	
4911	725.22736	[M + H]+	n.d.	C33H40O18	Melitidin	Flavonoid-7-O-glycosides	Citrus fruits	Elhady et al., 2024
4914	314.13849	[M + H]+	[M-H]-	C18H19NO4	N-cis-Feruloyltyramine	Hydroxycinnamic acids	*T. terrestris*	Uysal et al., 2024
4924	261.11182	[M-H2O + H]+	n.d.	C15H18O5	Meranzin hydrate	Coumarins - antidepressant properties	Citrus fruits	Tsujimoto et al., 2018
5008	284.12775	[M + H]+	[M-H]-	C17H17NO3	Coumaroyl tyramine	Hydroxycinnamic acids	*T. terrestris*	Uysal et al., 2023
5054	1065.5457	[M + H-H2O]+	n.d.	C51H86O24	Terrestrosin H	Steroidal saponins	*T. terrestris*	Wang et al., 2025
5072	314.13803	[M + H]+	[M-H]-	C18H19NO4	N-trans-Feruloyltyramine	Hydroxycinnamic acids	*T. terrestris*	Uysal et al., 2023
5.142	1031.5404	[M + H-H2O]+	n.d.	C51H84O24	Protodioscin	Steroidal saponins	*T. terrestris*	Wang et al., 2024
5158	1033.5536	[M + H-H2O]+	n.d.	C51H86O22	Neoprotodioscin	Steroidal saponins	*T. terrestris*	Wang et al., 2024
5197	305.10165	[M + H]+	[M + FA-H]-	C16H16O6	Heraclenol	Coumarins	Citrus fruits	Lü et al., 2022
5.258	475.2117	[M + H]+	n.d.	C22H30N6O4S	Sildenafil	Phosphodiesterase-5 inhibitors	Analytical standard	
5275	1313.6351	[M + H-H2O]+	[M-H]-	C61H102O31	Terrestrinin B	Steroidal saponins	*T. terrestris*	Wang et al., 2027
5276	965.4397	[M + H-H2O]+	n.d.	C45H74O21S	Prototribestin	Steroidal saponins	*T. terrestris*	Wang et al., 2024
5613	273.07538	[M + H]+	[M-H]-	C15H12O5	Naringenin	Flavanones	Citrus fruits	Elhady et al., 2024
5654	261.11191	[M + H]+	n.d.	C15H16O4	Auraptenol	Coumarins - antidepressant properties	Citrus fruits	Nakatani et al., 1987;Yang et al., 2019
5834	333.16919	[M + H]+	[M + FA-H]-	C19H24O5	Marmin	Coumarins	Citrus fruits	Lü et al., 2022
5949	625.25342	[M + H]+	n.d.	C36H36N2O8	Tribulusamide B	Lignanamides	*T. terrestris*	Uysal et al., 2023
6011	390.14478	[M + H]+	n.d.	C22H19N3O4	Tadalafil	Phosphodiesterase-5 inhibitors	Analytical standard	
6102	261.11185	[M + H]+	n.d.	C15H16O4	Meranzin	Coumarins - antidepressant properties	Citrus fruits	Tsujimoto et al., 2019
6.567	287.2005	[M + H]+	n.d.	C19H26O2	4-Androstene-3,17-dione	Steroid anabolics	Analytical standard	
7292	415.32062	[M + H]+	n.d.	C27H42O3	Diosgenin	Steroid sapogenins	*T. terrestris*	Uysal et al., 2023
7293	869.48871	[M + H]+	n.d.	C45H72O16	Dioscin	Steroidal saponins	*T. terrestris*	Wang et al., 2024
8073	427.28415	[M + H]+	n.d.	C27H38O4	Spirost-4-ene-3,12-dion	Steroid sapogenins	*T. terrestris*	Wang et al., 2024
8417	345.24237	[M + H]+	n.d.	C22H32O3	Testosterone propionate	Steroid anabolics	Analytical standard	

### 3.2 Discriminating between authentic and simulated fraudulent *T. terrestris* extracts

The LC-HRMS metabolomics profiles of authentic plant samples and simulated fraudulent samples of *T. terrestris* were subjected to principal component analysis (PCA) as a pattern recognition and unsupervised variable reduction technique.

**Fig. 1:**
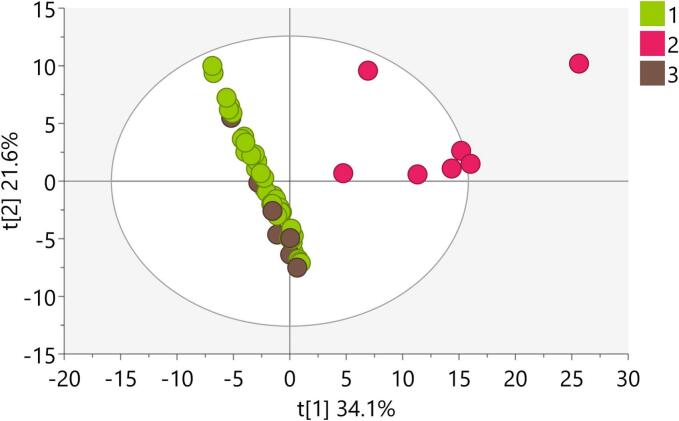
Score plot of PCA model containing: 1-Authentic samples of *T. terrestris*, 2- Authentic samples of *T. terrestris* spiked with “PDE5i mix”, 3- Authentic samples of *T. terrestris* spiked with “Steroid mix”.

**Fig. 2:**
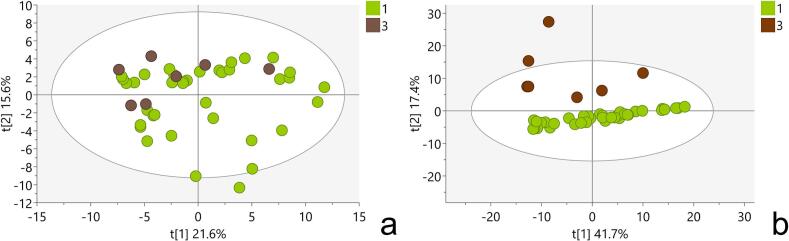
a) Score plot of PCA model M2 containing: 1-Authentic samples of *T. terrestris*, 3- Authentic samples of *T. terrestris* spiked with “Steroid mix” b) Score plot of PCA model M3 created with refined dataset containing only steroid-like features: 1-Authentic samples of *T. terrestris*, 3- Authentic samples of *T. terrestris* spiked with “Steroid mix”.

The PCA score plots of the first two principal components showed a clear separation of the samples spiked with PDE5i mix from the other samples (model M1, Table S4, Fig. 1). The score contribution plot (Fig. S1) showed that vardenafil, tadalafil, and sildenafil were responsible for this separation. The results indicated that the PCA model used can identify probable adulteration of *T. terrestris*-based preparations with these three chemicals, all of which were the constituents of the PDE5i mixture. No obvious clustering between authentic and simulated fraudulent *T. terrestris* samples spiked with the Steroid mix was detected in PCA model M1. The lack of separation on the score plot was still evident when a new PCA model (M2) omitted samples spiked with the PDE5i mix (Table S4, Fig. 2a). To overcome these difficulties, a new PCA model (M3) was created using a refined dataset containing only steroid-like features. These features were extracted by SteroidXtract, a convolutional neural network (CNN)-based tool that recognizes steroid-like features based on their unique MS/MS pattern (Xing et al., 2021). Indeed, a separation between authentic and simulated fraudulent *T. terrestris* samples spiked with the Steroid mix is now clearly visible (Table S4, Fig. 2b). The score contribution plot (Fig. S2) showed that testosterone propionate and 4-androstene-3,17-dione, the constituents of the Steroid mix, were responsible for this separation. This indicates that refining datasets to contain steroid-like features only can lead to easier detection of adulteration of *T. terrestris*-based preparations with anabolic steroids.

### 3.3 Revealing adulteration and authenticity in commercial *T. terrestris* products

**Fig. 3:**
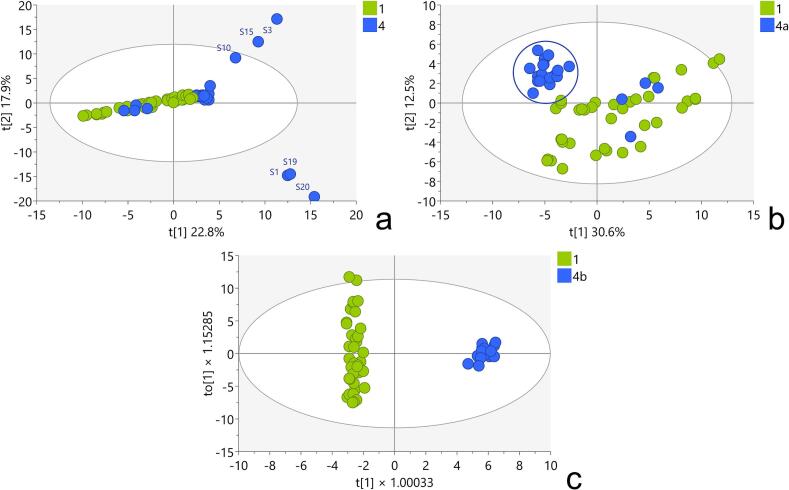
a) Score plot of PCA model M4 containing: 1-Authentic samples of *T. terrestris*, 4- commercially obtained products; Fig. 4. b) Score plot of PCA model M5 containing: 1-Authentic samples of *T. terrestris*, 4a- commercially obtained products excluding outliers from model M4; c) Score plot of OPLS-DA model (M6) containing: 1-Authentic samples of *T. terrestris*, 4b- commercially obtained products appearing as a separate group in PCA model M5.

Next, a PCA model (M4) was created using authentic plant samples and commercially obtained products based on *T. terrestris*. In the score plot of the first two principal components of this model, two distinct groups of supplements deviated significantly from the central cluster, with samples S3, S10, S15, S19, and S20 identified as outliers (Table S4, Fig. 3a). The contribution plots of the PCA model, including samples S3, S10, and S15, were examined to identify the variables primarily responsible for the observed deviations. In all three instances, caffeine had the greatest contribution, followed by catechin tannins, *i.e.*, gallocatechin gallate, gallocatechin, epicatechin gallate, and epicatechin, and the flavonoid vitexin-2′-rhamnoside (Figs. S3-S5). Since these metabolites are characteristic of green tea (*Camellia sinensis*) (Morikawa et al., 2013), their presence strongly indicates that supplements S3, S10, and S15 were adulterated with green tea extracts. It was reported that many herbal dietary supplements contain green tea, although their presence may not always be mentioned on the product label (Navarro et al., 2013).

Producers may incorporate green tea to boost the stimulant properties of the supplement, appealing to consumers seeking increased energy, alertness, or improved athletic performance (Grgic et al., 2020). Compounds in green tea, particularly caffeine and catechin tannins, possess potent antioxidant effects and may enhance metabolism, fat oxidation, and endurance, making the supplement appear more efficacious (Meyer et al., 2023). Green tea extracts are widely available and cost-effective, allowing manufacturers to reduce production costs while retaining some properties of a *T. terrestris* supplement. Furthermore, it is crucial to recognize that the adulteration of supplements with green tea extracts may pose considerable safety risks. Numerous reported cases have documented significant hepatitis and liver damage, with symptoms improving upon discontinuation of the supplement (Navarro et al., 2013). This underscores the need for stringent analytical techniques, such as the untargeted LC-HRMS metabolomics method used in this study, to accurately identify and characterize adulterants, thereby ensuring product authenticity and customer safety (Navarro et al., 2013).

The contribution plots of PCA model M4 for samples S1, S19, and S20 revealed that the predominant contributors were meranzin and meranzin hydrate, followed by other coumarins (heraclenol, marmin, and auraptenol) and flavonoids (naringin, naringenin, prunin, and melitidin) (Figs. S6-S8). These compounds are commonly found in citrus peels (Nakatani et al., 1987; Tsujimoto et al., 2018; Lü et al., 2022; Elhady et al., 2024), indicating that the supplements have been adulterated with citrus-derived components. Further HR-LCMS analysis, conducted without prior clean-up using an SPE cartridge, revealed the presence of synephrine and *N*-methyltyramine in S1, S19, and S20. However, synephrine levels ranged from approximately 7 to 90 ppm, which is significantly lower than those detected in the positive control—a supplement explicitly labeled as containing bitter orange (*Citrus aurantium*) extract with 1.25 % synephrine. In contrast, the negative control (S4), a randomly selected supplement with no declared citrus peel content, did not contain these compounds (Supplementary Material, Text S1, Table S5).

The presence of *N*-methylated tyramine in citrus plants, alongside synephrine, has been well documented in the literature (Servillo et al., 2017). Additionally, synephrine is widely recognized as a bioactive compound in *C. aurantium* and is found in smaller amounts in other citrus species, often associated with weight loss and performance enhancement (Esposito et al., 2023).

After removing the outliers from model M4, a second PCA model (M5, Table S4, Fig. 3b) was constructed to provide a better picture of the variation within the central cluster in model M4. This refined model revealed unique subclusters among the commercial products. Four samples were clustered with the authentic *T. terrestris* samples in the PCA score plot, but the other commercial products formed a distinct group.

An orthogonal partial least squares discriminant analysis (OPLS-DA) model was developed (M6, Table S4, Fig. 3c) to examine the compositional variances between authentic *T. terrestris* samples and commercial products. The four commercial products that were initially clustered with authentic samples in PCA model M5 were excluded from the OPLS-DA model to focus on the most divergent subgroup. Unlike PCA, OPLS-DA is a supervised method that increases group differentiation and identifies the variables primarily responsible for the observed differences. This targeted analysis allows a more accurate identification of the metabolites responsible for the compositional differences between authentic samples and the divergent subgroup of commercial products.

As expected, the commercial products (subgroup 4b) that were separated in PCA model M5 were now distinctly separated from the authentic *T. terrestris* samples in the OPLS-DA model. The S-plot of the OPLS-DA model was examined to identify the metabolites primarily responsible for the differentiation between the groups (Fig. S9). In the S-plot, variables located at the extremes represent the most significant chemicals differentiating the two groups. In a segment of the S-plot pertaining to subgroup 4b, terrestrinin U was identified as the predominant distinguishing metabolite, whereas the opposite segment associated with authentic *T. terrestris* samples revealed protodioscin, rutin, neoprotodioscin, prototribestin, and N-trans-feruloyltyramine as the key distinguishing metabolites. All of these metabolites were thoroughly documented constituents of *T. terrestris*, confirming the authenticity of the phytochemical profiles (Uysal et al., 2023; Wang et al., 2024). Compositional variations between authentic *T. terrestris* and specific commercial products can arise from natural variations in chemical composition, which are influenced by ecological factors.

A PCA model (M7) was ultimately constructed containing authentic *T. terrestris* plant samples and commercially obtained products, refined using the SteroidXtract tool. This dataset included only steroid-like features, such as steroidal saponins and other steroidal compounds commonly found in *T. terrestris*, as well as anabolic steroids from commercial products that could indicate possible adulteration.

**Fig. 4:**
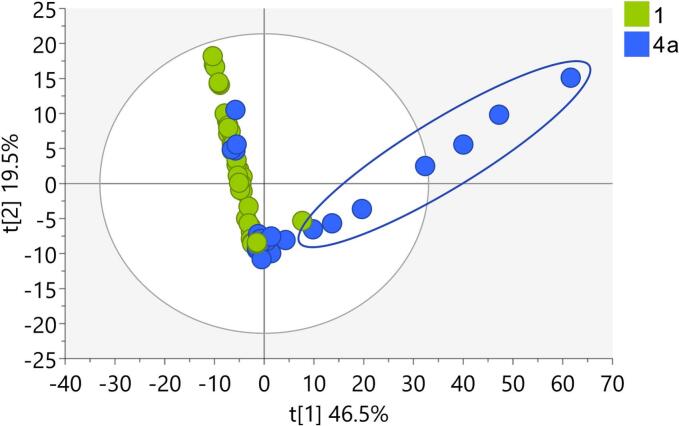
Score plot of PCA model M7 containing: 1-Authentic samples of *T. terrestris*, 4a- commercially obtained products excluding outliers from model M4.

The score plot of the first two principal components revealed that all authentic samples and most commercial products were in the central cluster. Seven commercial products exhibited a distinct pattern resembling a linear regression line (Table S4, Fig. 4). Contribution plots indicated spirost-4-ene-3,12-dione as the principal distinguishing element (Fig. S10). The greater the deviation of the scores from the central cluster, the higher the concentration of this component in the respective samples. Spirost-4-ene-3,12-dione is known to potentially form from the degradation of more complex steroidal saponins, as observed during the stir-frying of *T. terrestris* fruits (Wang et al., 2024). Although environmental factors such as high temperature, prolonged storage, light exposure, and humidity have been implicated in accelerating these degradation processes (Wu et al., 2024), further research is needed to fully explain these mechanisms in commercial products. Importantly, no contamination with anabolic steroids was detected in the analyzed supplements.

In contrast to prior research that concentrated on the authentication of *T. terrestris*-based products through chromatographic fingerprinting (Custers et al., 2017) or the targeted identification of PDE5 inhibitors in herbal supplements (Jiru et al., 2019), our untargeted LC-HRMS metabolomics methodology provides a more comprehensive examination. Although chromatographic fingerprinting techniques are efficient in verifying the presence of declared botanical ingredients, they are limited in detecting unexpected adulterants. Similarly, targeted analyses have excellent sensitivity for specific compounds but are restricted to detecting only a predetermined set of adulterants. Our methodology, which combines high-resolution mass spectrometry with multivariate statistical analysis and CNN-based filtering, not only authenticates *T. terrestris*-based products but also identifies a wide range of both expected and unexpected adulterants, thereby improving the overall safety evaluation of these products.

### 3.4 Conclusion remarks

This study effectively utilized an untargeted LC-HRMS metabolomics approach to assess the authenticity and potential adulteration of dietary supplements derived from *T. terrestris*. Through multivariate data analysis, the existence of PDE5 inhibitors (sildenafil, vardenafil, and tadalafil) and anabolic steroids (testosterone propionate and 4-androstene-3,17-dione) in spiked samples was confirmed, with refined feature selection *via* the SteroidXtract tool augmenting detection precision. This method offers a more reliable detection of adulteration in *T. terrestris*-based formulations, particularly in identifying anabolic steroids and PDE5 inhibitors, thereby enhancing consumer safety.

PCA analysis further revealed that certain commercial products contained non-declared unexpected constituents, such as green tea and citrus-derived substances, likely added to enhance stimulant effects or modify hormone-related characteristics. The identification of spirost-4-ene-3,12-dione in specific products suggests the potential degradation of steroidal saponins, probably due to inappropriate storage or processing conditions.

Compared to spectroscopic techniques like FTIR and hyperspectral imaging, the untargeted LC-HRMS metabolomics method presented in this study offers superior sensitivity, specificity, and chemical resolution. While spectroscopic approaches are fast and non-destructive, they often face challenges in detecting trace-level or structurally similar adulterants. In contrast, LC-HRMS enables precise identification of molecules in complex mixtures, thanks to its high mass accuracy and detailed fragmentation patterns.

In contrast to NMR, which requires expensive equipment and has reduced sensitivity for trace-level detection, LC-HRMS offers enhanced sensitivity and covers a wider range of molecules. Although PCR-based techniques are efficient in detecting DNA-containing adulterants, they are inadequate for identifying synthetic compounds or non-biological adulterants. The combination of metabolomics with multivariate data analysis significantly improves the reliability of adulteration detection, offering a more robust and comprehensive approach to verifying the authenticity and safety of dietary supplements.

In summary, our untargeted LC-HRMS metabolomics methodology offers considerable advantages over current techniques in detecting adulteration in *T. terrestris*-based products. Unlike chromatographic fingerprinting approaches, which primarily focus on plant identification, our method can detect a broader spectrum of adulterants. Similarly, while targeted LC-HRMS techniques offer exceptional sensitivity for known PDE5 inhibitors, they are intrinsically limited to predefined analyte lists. The comprehensive detection capability of our method, along with sophisticated chemometric techniques, ensures authenticity verification and effective adulteration screening, marking a significant improvement in the quality control of herbal dietary supplements.

These findings underscore the importance of comprehensive metabolomics analysis in evaluating the authenticity of herbal dietary supplements and identifying potentially fraudulent activities. The research emphasizes the need for stringent quality control and regulatory protocols to prevent the distribution of adulterated products and safeguard consumer safety.

## CRediT authorship contribution statement

**Dejan Gođevac:** Writing – review & editing, Writing – original draft, Supervision, Resources, Investigation, Funding acquisition, Data curation, Conceptualization. **Jovana Stanković Jeremić:** Investigation, Formal analysis. **Mirjana Cvetković:** Writing – original draft, Formal analysis. **Katarina Simić:** Project administration, Formal analysis. **Ivana Sofrenić:** Investigation. **Jovana Ljujić:** Formal analysis. **Lazar Popović:** Software, Data curation. **Uroš Gašić:** Writing – original draft, Formal analysis. **Yen-Nhi Hoang:** Data curation. **Tao Huan:** Writing – review & editing, Software, Methodology. **Stefan Ivanović:** Writing – original draft, Visualization, Methodology, Investigation.

## Declaration of competing interest

The authors declare that they have no known competing financial interests or personal relationships that could have appeared to influence the work reported in this paper.

## Data Availability

Data will be made available on request.

## Appendix A Supplementary data

Supplementary material: Contains detailed data and analyses supporting the manuscript. Image 1

## References

[bb0005] Blaženović I., Kind T., Ji J., Fiehn O. (2018). Software tools and approaches for compound identification of LC-MS/MS data in metabolomics. Metabolites.

[bb0010] Chen Z., Lian X., Zhou M., Zhang X., Wang C. (2023). Quantitation of L-cystine in food supplements and additives using ^1^H qNMR: Method development and application. Foods.

[bb0015] Custers D., Van Praag N., Courselle P., Apers S., Deconinck E. (2017). Chromatographic fingerprinting as a strategy to identify regulated plants in illegal herbal supplements. Talanta.

[bb0020] Elhady S.S., Youssef F.S., Lashkar M.O., Hamdan D.I., Ashour M.L., Zengin G., Gamal E.-D.M.I. (2024). Chemometric discrimination of eight citrus plants utilizing chromatographic and spectroscopic techniques and insights into their biological potentials. Current Research in Food Science.

[bb0025] Esposito G., Sciuto S., Martello E., Pezzolato M., Bozzetta E. (2023). Disclosing frauds in herbal food supplements labeling: A simple LC-MS/MS approach to detect alkaloids and biogenic amines. Journal of Food Protection.

[bb0030] Ghosh V.K., Bhope S.G., Kuber V.V., Sagulale A.D. (2012). An improved method for the exrction and quantitation of diosgenin in *Tribulus terrestris* L. Journal of Liquid Chromatography and Related Technologies.

[bb0035] Gloyn A., Faber J., Malmodin D., Thanabalasingham G., Lam F., Ueland P., Baunsgaard D. (2012). Metabolic profiling in maturity-onset diabetes of the young (MODY) and young onset type 2 diabetes fails to detect robust urinary biomarkers. PLoS One.

[bb0040] Grgic J., Grgic I., Pickering C., Schoenfeld B.J., Bishop D.J., Pedisic Z. (2020). Wake up and smell the coffee: Caffeine supplementation and exercise performance—An umbrella review of 21 published meta-analyses. British Journal of Sports Medicine.

[bb0045] Guo J., Yu H., Xing S., Huan T. (2022). Addressing big data challenges in mass spectrometry-based metabolomics. Chemical Communications.

[bb0050] Hosseini E., Ghasemi J.B., Shekarchi M. (2022). Simultaneous determination of adulterants in dietary food supplements using multivariate data analysis after preconcentration with novel nanosorbents and chromatographic measurement. Journal of AOAC International.

[bb0055] Jiang D.S.Y., Dong K., Zhao K., Liu S., Qu B., Fu S., Zhao F. (2024). Research advances in detection of food adulteration and application of MALDI-TOF MS: A review. Food Chemistry.

[bb0060] Jiru M., Stranska-Zachariasova M., Dzuman Z., Hurkova K., Tomaniova M., Stepan R., Hajslov J. (2019). Analysis of phosphodiesterase type 5 inhibitors as possible adulterants of botanical-based dietary supplements: Extensive survey of preparations available at the Czech market. Journal of Pharmaceutical and Biomedical Analysis.

[bb0065] Kioukia-Fougia N., Georgiadis N., Tsarouhas K., Vasilaki F., Fragkiadaki P., Meimeti E., Tsitsimpikou C. (2016). Synthetic and natural nutritional supplements: Health “allies” or risks to public health?. Recent Patents on Inflammation & Allergy Drug Discovery.

[bb0075] Lü J., Zhang D., Zhang X., Sa R., Wang X., Wu H., Zhang B. (2022). Network analysis of the herb–drug interactions of citrus herbs inspired by the “Grapefruit Juice Effect”. ACS Omega.

[bb0080] Lv D., Wang D., Li D., Guo D., Qi M., Zhang Y., Chai Y., Chen X., Cao Y. (2024). A novel standard-free detection of adulteration method for sildenafil derivatives in dietary supplements. Biomedical Chromatography.

[bb0085] Meyer B.R., White H.M., McCormack J.D., Niemeyer E.D. (2023). Catechin composition, phenolic content, and antioxidant properties of commercially-available bagged, gunpowder, and Matcha green teas. Plant Foods for Human Nutrition.

[bb0090] Morikawa T., Ninomiya K., Miyake S., Miki Y., Okamoto M., Yoshikawa M., Muraoka O. (2013). Flavonol glycosides with lipid accumulation inhibitory activity and simultaneous quantitative analysis of 15 polyphenols and caffeine in the flower buds of *Camellia sinensis* from different regions by LCMS. Food Chemistry.

[bb0095] Nakatani N., Yamada Y., Fuwa H. (1987). 7-Geranyloxycoumarin from juice oil of Hassaku (Citrus hassaku) and antimicrobial effects of related Coumarins. Agricultural and Biological Chemistry.

[bb0100] Navarro V.J., Bonkovsky H.L., Hwang S.I., Vega M., Barnhart H., Serrano J. (2013). Catechins in dietary supplements and hepatotoxicity. Digestive Diseases and Sciences.

[bb0105] Pujol C., Danoun S., Biasini G., Retailleau E., Masson J., Balayssac S., Gilard V. (2024). Benchtop NMR coupledwith chemometrics: A workflow for unveiling hidden drug ingredients in honey-based supplements. Molecules.

[bb0110] Roiffé R.R., Sardela V.F., Lima A.L.S., Oliveira D.S., Aquino Neto F.R., Lima K.S.C., de la Cruz M.N.S. (2019). Determination of adulterants in whey protein food supplements by liquid chromatography coupled to Orbitrap high resolution mass spectrometry. Brazilian Journal of Food Technology.

[bb0115] Saeed M., Munawar M., Bi J., Ahmed S., Ahmad M.Z., Ali Kamboh A., Chen H. (2024). Promising phytopharmacology, nutritional potential, health benefits, and traditional usage of *Tribulus terrestris* L. herb. Heliyon.

[bb0120] Servillo L., Castaldo D., Giovane A., Casale R., D’Onofrio N., Cautela D., Balestrieri M.L. (2017). Tyramine pathways in Citrus plant defense: Glycoconjugates of tyramine and its N-methylated derivatives. Journal of Agricultural and Food Chemistry.

[bb0125] Sota-Uba I., Bamidele M., Moulton J., Booksh K., Lavine B.K. (2021). Authentication of edible oils using Fourier transform infrared spectroscopy and pattern recognition methods. Chemometrics and Intelligent Laboratory Systems.

[bb0130] Stefanescu R., Laczko-Zold E., Mare A., Esianu S., Grama I., Negroiu A., Vari C. (2020). Risks and benefits associated with *Tribulus terrestris* products assessed by phytochemical and pharmacological screening. Revista de Chimie.

[bb0135] Taghvimi A., Hamidi S., Nemati M. (2019). Magnetic dispersive solid phase microextraction technique coupled with LC–MS/MS for evaluating content versus label claims in ephedrine-free food supplements. Journal of Consumer Protection and Food Safety.

[bb0140] Trbović D., Petronijević R., Đorđević V. (2017). Chromatography methods and chemometrics for determination of milk fat adulterants. IOP Conference Series: Earth and Environmental Science.

[bb0145] Tsugawa H., Cajka T., Kind T., Ma Y., Higgins B., Ikeda K., Arita M. (2015). MS-DIAL: Data-independent MS/MS deconvolution for comprehensive metabolome analysis. Nature Methods.

[bb0150] Tsugawa H., Kind T., Nakabayashi R., Yukihira D., Tanaka W., Cajka T., Arita M. (2016). Hydrogen rearrangement rules: Computational MS/MS fragmentation and structure elucidation using MS-FINDER software. Analytical Chemistry.

[bb0155] Tsujimoto T., Yoshitomi T., Maruyama T., Yamamoto Y., Hakamatsuka T., Uchiyama N. (2019). High-resolution liquid chromatography–mass spectrometry-based metabolomic discrimination of citrus-type crude drugs and comparison with nuclear magnetic resonance spectroscopy-based metabolomics. Journal of Natural Products.

[bb0160] Uysal S., Senkardes I., Jekő J., Cziáky Z., Zengin G. (2023). Chemical characterization and pharmacological profile of *Tribulus terrestris* extracts: A novel source of cosmeceuticals and pharmaceuticals. Biochemical Systematics and Ecology.

[bb0165] Walker K.M., Applequist W.L. (2012). Adulteration of selected unprocessed botanicals in the U.S. retail herbal trade. Economic Botany.

[bb0170] Wallace E.D., Todd D.A., Harnly J.M., Cech N.B., Kellogg J.J. (2020). Identification of adulteration in botanical samples with untargeted metabolomics. Analytical and Bioanalytical Chemistry.

[bb0175] Wang S., Du D.F., Li F., Chen M.Y., Sheng H.G., Zhang C., Guo F., Chen Z., Cao G.S. (2024). "UHPLC-Q-TOF/MS-chemometrics-network pharmacology" integrated strategy to discover quality markers of raw and stir-fried Fructus Tribuli and process optimization of stir-fried Fructus Tribuli. Phytochemical Analysis.

[bb0180] Wu Y., Zheng H., Zheng T., Jiang J., Xu Y., Jia F., Yang Y. (2024). Quantitative changes and transformation mechanisms of Saponin components in Chinese herbal medicines during storage and processing: A review. Molecules.

[bb0185] Xie J., Pan Q., Li F., Tang Y., Hou S., Xu C. (2021). Simultaneous detection of trace adulterants in food based on multi-molecular infrared (MM-IR) spectroscopy. Talanta.

[bb0190] Xing S., Jiao Y., Salehzadeh M., Soma K.K., Huan T. (2021). SteroidXtract: Deep learning-based pattern recognition enables comprehensive and rapid extraction of steroid-like metabolic features for automated biology-driven metabolomics. Analytical Chemistry.

[bb0195] Yang C.-S., Han S.-Q., Wang X., Zhou T., Dong X.-Y., Bo P. (2019). RRLC-DAD-ESI-MS based and bioactivity guided phytochemical analysis and separation of coumarins from raw extracts of Trigonostemon lutescens. Journal of Pharmaceutical and Biomedical Analysis.

[bb0200] Zahedi R., Eghlima G., Mirjalili M.H., Aliahmadi A., Esmaeili G. (2024). Diosgenin content, phenolic acids, and antioxidant activity of different parts of Iranian *Tribulus terrestris* L. Genetic Resources and Crop Evolution.

