# Supplementary material for: LC-MS-based metabolomics for detecting adulteration in Tribulus terrestris-derived dietary supplements
Source: Food Chem X. 2025 Apr 18;27:102476. doi: 10.1016/j.fochx.2025.102476 (PMC12033958; doi:10.1016/j.fochx.2025.102476)
Supplement: Supplementary file 1 — Supplementary material: Contains detailed data and analyses supporting the manuscript. [file mmc1.docx]

**LC-MS-based metabolomics for detecting adulteration in *Tribulus terrestris*-derived dietary supplements**

Dejan Gođevac^a^*, Jovana Stanković Jeremić^a^, Mirjana Cvetković^a^, Katarina Simić^a^, Ivana Sofrenić^b^, Jovana Ljujić^b^, Lazar Popović^c^, Uroš Gašić^d^, Yen-Nhi Hoang^e^, Tao Huan^e^, Stefan Ivanović^a^

Affiliations:

^a^ University of Belgrade, Institute of Chemistry, Technology and Metallurgy ‒ National Institute of the Republic of Serbia, Njegoševa 12, 11000 Belgrade, Serbia

^b^ University of Belgrade – Faculty of Chemistry, Studentski trg 12-16, 11000 Belgrade, Serbia

^c^ University of Belgrade – Innovation Center of Faculty of Chemistry, Studentski trg 16, 11000 Belgrade, Serbia

^d^ University of Belgrade – Institute for Biological Research "Siniša Stanković" ‒ National Institute of Republic of Serbia, Bulevar Despota Stefana 142, 11000 Belgrade, Serbia

^e^ Department of Chemistry, Faculty of Science, University of British Columbia, Vancouver Campus, 2036 Main Mall, Vancouver, BC V6T 1Z1, Canada

*Corresponding author.

*E-mail address*: dejan.godjevac@ihtm.bg.ac.rs (D. Gođevac).

Table S1. Collection data of *T. terrestris* samples

| No. | Country of collection | Collection site | GPS coordinate | Year of collecting | Month of collecting | Plant part | Voucher specimen No. |
| --- | --- | --- | --- | --- | --- | --- | --- |
| 1 | Bulgaria | Asenovgrad | Latitude: 42.0074; Longitude: 24.8690 | 2024 | September | Leaves, stems, fruits | S71/24 |
| 2 | Bulgaria | Asenovgrad | Latitude: 42.0074; Longitude: 24.8690 | 2024 | September | Leaves, stems, fruits | S71/24 |
| 3 | Bulgaria | Asenovgrad | Latitude: 42.0074; Longitude: 24.8690 | 2024 | September | Fruits | S71/24 |
| 4 | Greece | Palio | Latitude: 40.9054; Longitude: 24.3477 | 2024 | September | Leaves, stems, fruits | S72/24 |
| 5 | Greece | Palio | Latitude: 40.9054; Longitude: 24.3477 | 2024 | September | Leaves, stems, fruits | S72/24 |
| 6 | Greece | Palio | Latitude: 40.9054; Longitude: 24.3477 | 2024 | September | Leaves, stems, fruits | S72/24 |
| 7 | Greece | Palio | Latitude: 40.9054; Longitude: 24.3477 | 2024 | September | Fruits | S72/24 |
| 8 | Greece | Krinides | Latitude: 41.0125; Longitude: 24.2901 | 2024 | September | Leaves, stems, fruits | S73/24 |
| 9 | Greece | Krinides | Latitude: 41.0125; Longitude: 24.2901 | 2024 | September | Fruits | S73/24 |
| 10 | Serbia | Novi Beograd | Latitude: 44.7963; Longitude: 20.3901 | 2023 | Oktober | Leaves, stems, fruits | S74/24 |
| 11 | Serbia | Novi Beograd | Latitude: 44.7963; Longitude: 20.3901 | 2023 | Oktober | Fruits | S74/24 |
| 12 | Serbia | Kalemegdan | Latitude: 44.8233; Longitude: 20.4508 | 2024 | August | Leaves, stems, fruits | S75/24 |
| 13 | Serbia | Kalemegdan | Latitude: 44.8233; Longitude: 20.4508 | 2024 | August | Leaves, stems, fruits | S75/24 |
| 14 | Serbia | Kalemegdan | Latitude: 44.8233; Longitude: 20.4508 | 2024 | August | Fruits | S75/24 |
| 15 | Serbia | Novi Beograd | Latitude: 44.7963; Longitude: 20.3901 | 2024 | Oktober | Leaves, stems, fruits | S76/24 |
| 16 | Serbia | Novi Beograd | Latitude: 44.7963; Longitude: 20.3901 | 2024 | Oktober | Fruits | S76/24 |
| 17 | Serbia | Novi Beograd | Latitude: 44.7963; Longitude: 20.3901 | 2024 | Oktober | Fruits | S76/24 |
| 18 | Serbia | Novi Beograd | Latitude: 44.7963; Longitude: 20.3901 | 2024 | Oktober | Fruits | S76/24 |
| 19 | Serbia | Zemun | Latitude: 44.8419; Longitude: 20.4101 | 2021 | August | Leaves, stems, fruits | S77/24 |
| 20 | Serbia | Zemun | Latitude: 44.8419; Longitude: 20.4101 | 2021 | August | Leaves, stems, fruits | S77/24 |
| 21 | Serbia | Zemun | Latitude: 44.8419; Longitude: 20.4101 | 2021 | August | Leaves, stems, fruits | S77/24 |
| 22 | Bulgaria | Burgas | Latitude: 42.5083; Longitude: 27.4837 | 2021 | August | Leaves, stems, fruits | S78/24 |
| 23 | Bulgaria | Burgas | Latitude: 42.5083; Longitude: 27.4837 | 2021 | August | Leaves, stems, fruits | S78/24 |
| 24 | Bulgaria | Burgas | Latitude: 42.5083; Longitude: 27.4837 | 2021 | August | Leaves, stems, fruits | S78/24 |
| 25 | Serbia | Zaječar | Latitude: 43.9064; Longitude: 22.2818 | 2019 | August | Leaves, stems, fruits | S79/24 |
| 26 | Serbia | Zaječar | Latitude: 43.9064; Longitude: 22.2818 | 2019 | August | Leaves, stems, fruits | S79/24 |
| 27 | Serbia | Zaječar | Latitude: 43.9064; Longitude: 22.2818 | 2019 | August | Leaves, stems, fruits | S79/24 |
| 28 | Serbia | Zaječar | Latitude: 43.9064; Longitude: 22.2821 | 2020 | September | Leaves, stems, fruits | S80/24 |
| 29 | Serbia | Zaječar | Latitude: 43.9064; Longitude: 22.2821 | 2020 | September | Leaves, stems, fruits | S80/24 |
| 30 | Serbia | Zaječar | Latitude: 43.9064; Longitude: 22.2821 | 2020 | September | Leaves, stems, fruits | S80/24 |
| 31 | Greece | Thessaloniki | Latitude: 40.6443; Longitude: 22.9407 | 2021 | September | Leaves, stems, fruits | S81/24 |
| 32 | Greece | Thessaloniki | Latitude: 40.6443; Longitude: 22.9407 | 2021 | September | Leaves, stems, fruits | S81/24 |
| 33 | Greece | Thessaloniki | Latitude: 40.6443; Longitude: 22.9407 | 2021 | September | Leaves, stems, fruits | S81/24 |
| 34 | Serbia | Smederevo | Latitude: 44.6676; Longitude: 20.9278 | 2024 | September | Leaves, stems, fruits | S82/24 |
| 35 | Serbia | Smederevo | Latitude: 44.6676; Longitude: 20.9278 | 2024 | September | Fruits | S82/24 |
| 36 | Turkmenistan | Turkmenabat | Agro Industrial Complex Buyan | 2024 | Oktober | Fruits | - |
| 37 | Turkmenistan | Turkmenabat | Agro Industrial Complex Buyan | 2024 | Oktober | Fruits | - |
| 38 | Turkmenistan | Turkmenabat | Agro Industrial Complex Buyan | 2024 | Oktober | Fruits | - |

Table S2. Data of commercially obtained supplements

| **Supplement label** | **Country of manufacturing** | **Pharmaceutical form** | **Declared content of steroid saponins** | **Declared active ingedient** | **Other declared ingredients** |
| --- | --- | --- | --- | --- | --- |
| S1 | EU | capsules | 90% | *T. terrestris* plant extract | Not declared |
| S2 | Hungary | tablets | 24% | *T. terrestris* fruit extract | Microcrystalline cellulose, calcium phosphate, silicon dioxide, magnesium salts of fatty acids, hydroxypropyl methylcellulose, hydroxypropylcellulose, modified starch, triglycerides, talc, polyethylene glycol |
| S3 | Hungary | tablets | 40% | *T. terrestris* fruit extract | Maltodextrin, microcrystalline cellulose, hydroxypropyl methylcellulose, talc, silicon dioxide, magnesium stearate |
| S4 | Slovenia | capsules | 90% | *T. terrestris* fruit extract | Magnesium stearate |
| S5 | USA | capsules | 40% | *T. terrestris* plant and fruit | Dicalcium phosphate, magnesium stearate |
| S6 | UK | capsules | 90% | *T. terrestris* plant extract | Magnesium stearate, silicon dioxide, titanium dioxide, color |
| S7 | Slovenia | capsules | Not declared | *T. terrestris* fruit extract | hydroxypropyl methylcellulose, color chlorophyllin |
| S8 | Czech Republic | capsules | 90% | *T. terrestris* fruit extract | iron oxide, magnesium stearate, silicon dioxide |
| S9 | USA | capsules | 95% | *T. terrestris* fruit extract | cellulose |
| S10 | Hungary | tablets | 90% | *T. terrestris* fruit extract | maltodextrin, cellulose, magnesium salts of fatty acids, talc, silicon dioxide, polyvinylpyrrolidone |
| S11 | Germany | capsules | 90% | *T. terrestris* plant extract | hydroxypropyl methylcellulose, magnesium salts of fatty acids, silicon dioxide, zinc citrate, calcium D-pantothenate |
| S12 | Bulgarska | capsules | Not declared | *T. terrestris* leaf extract | creatine, magnesium stearate, microcrystalline cellulose |
| S13 | UK | capsules | 95% | *T. terrestris* fruit extract | hydroxypropyl methylcellulose, L-arginine HCl, magnesium stearate, silicon dioxide |
| S14 | Hungary | tablets | 24% | *T. terrestris* fruit extract | microcrystalline cellulose, calcium phosphate, magnesium salts of fatty acids, silicon dioxide, hydroxypropyl methylcellulose, talc, hydroxypropylcellulose, modified starch, triglycerides |
| S15 | Spain | capsules | 90% | *T. terrestris* plant extract | zinc gluconate, microcrystalline cellulose, magnesium stearate, hydroxypropyl methylcellulose, color |
| S16 | Slovenia | capsules | 40% | *T. terrestris* fruit extract | Titanium dioxide, iron oxide |
| S17 | Poland | capsules | 90% | *T. terrestris* plant extract | microcrystalline cellulose, magnesium salts of fatty acids, dyes |
| S18 | Bulgarska | tincture | 36% | *T. terrestris* fruit extract | glycerin 59%, purified water, zinc citrate |
| S19 | Poland | capsules | 90% | *T. terrestris* fruit extract | microcrystalline cellulose, starch, silicon dioxide, magnesium salts of fatty acids |
| S20 | Germany | tablets | 80% | *T. terrestris* plant extract | zinc gluconate, pregelatinized modified starch, carboxymethyl cellulose, soy lecithin, stearic acid, glycerol, colors (titanium dioxide, carminic acid, riboflavin), color (apple, blackcurrant and radish concentrate), magnesium stearate, silicon dioxide |
| S21 | Czech Republic | capsules | 90% | *T. terrestris* fruit extract | iron oxide, shellac, magnesium stearate |
| S22 | Czech Republic | capsules | 40% | *T. terrestris* fruit extract | iron oxide, zinc gluconate, magnesium stearate, calcium D-pantothenate |
| S23 | Bulgaria | capsules | 45% | *T. terrestris* fruit extract | cellulose, dicalcium phosphate, magnesium stearate |
| S24 | Bulgaria | capsules | Not declared | *T. terrestris* plant extract | zinc oxide, sodium selenite, microcrystalline cellulose, magnesium stearate, calcium phosphate |
| S25 | Germany | capsules | 90% | *T. terrestris* fruit extract | hydroxypropyl methylcellulose |
| S26 | Germany | capsules | 20% | *T. terrestris* fruit extract | zinc citrate |
| S27 | Bulgaria | tablets | 50% | *T. terrestris* plant extract | microcrystalline cellulose, colloidal anhydrous silica, povidone K25, crospovidone, magnesium stearate, talc |

Table S3. LC-MS data including the mass ppm error and MS/MS fragments for the adulterants identified

| Average Rt(min) | Average Mz, pos. mode | Mass error, ppm | MS/MS spectrum pos. mode | Molecular formula | Compound name |
| --- | --- | --- | --- | --- | --- |
| 3.699 | 307.08154 | -0.62 | 51.34277:63527 55.57336:56777 58.06515:133178 59.07288:92431 60.04571:60072 60.73401:54916 76.01202:64427 95.04897:69655 98.85054:61109 127.03888:126044 135.04401:145801 139.03897:28840878 140.04250:101269 151.03908:1321382 153.05484:218659 163.03917:2128229 169.04948:1500156 176.14597:77731 181.04970:3285376 188.61607:59318 195.06474:477622 198.78613:60289 205.04942:249703 219.06561:66204 223.06033:940392 247.06003:546425 265.07068:189715 271.06113:68728 289.07098:431285 307.08194:245508 | C15H14O7 | Epigallocatechin |
| 3.797 | 195.08765 | -0.01 | 54.21947:516176 62.51238:522663 64.12329:461566 83.11057:548122 97.38888:562755 138.05305:1035005 138.06610:14912761 162.14807:480995 180.06435:580603 195.08760:288046432 196.09163:499245 | C8H10N4O2 | Caffeine |
| 4.061 | 291.0867 | 1.32 | 58.06515:167299 59.07295:100352 60.08074:20384 68.06760:5698 91.05437:13065 105.06977:19209 117.06978:53025 118.04133:18224 119.04877:11449 119.08521:7950 123.04404:394476 124.04784:5324 127.03886:8234 131.08548:12444 133.06500:20442 139.03900:867427 139.59911:6692 146.89827:5186 147.04419:103451 149.12022:8868 151.03912:27440 153.59666:5386 161.05992:6305 163.03851:16759 163.13573:7524 165.05476:189963 169.04947:22095 176.14351:117028 177.14680:14658 179.07007:9573 181.04958:17485 184.12276:9161 189.05453:6298 190.15886:7660 192.13808:27415 207.06532:112268 220.12875:5780 231.06519:5981 231.39789:5343 234.16899:5057 234.28435:5932 235.14418:5108 237.36768:5548 240.15799:6541 245.09241:60716 246.78267:6495 249.07578:21598 249.41870:5070 249.67043:7460 250.17007:11170 253.17902:8420 261.51685:6491 261.85437:6945 262.18213:10590 267.77451:8520 273.07693:23429 273.12451:8273 291.08575:47533 291.12976:11351 291.17432:7583 | C15H14O6 | Epicatechin |
| 4.675 | 581.18585 | -1.09 | 57.03354:386679 69.03345:223785 71.04913:3302214 75.04436:134690 83.04949:139625 85.02845:4482092 121.06479:284043 129.05455:1632090 147.04367:187373 147.06546:329667 153.01830:5162684 171.02885:517214 179.03415:258884 195.02890:1364542 219.02882:545507 219.09692:112623 226.38985:119152 231.02608:123586 245.04564:128978 260.27914:125219 261.03961:320621 263.05383:599681 273.07599:24213010 279.04919:191096 281.06412:220371 285.07474:744712 297.07303:439973 315.08673:675622 339.08752:1127142 351.08490:205150 357.47394:133384 365.10336:220735 381.09879:235964 383.11285:268725 399.10837:183913 453.78458:130125 492.69714:128009 | C27H32O14 | Naringin |
| 4.746 | 435.12817 | -0.93 | 56.15767:4413 58.06538:19626 59.07308:8541 62.91097:5242 73.66949:4393 74.15474:4913 75.31232:4772 77.36960:4793 85.02852:27020 97.02844:5160 105.12234:6236 117.07047:5765 127.03941:11224 147.04355:8297 153.01765:25832 167.07089:26269 176.14394:11068 181.04984:16786 181.08647:5877 189.05557:7243 221.08189:6838 226.39580:7710 233.08162:22269 243.10220:16542 261.11313:5455 273.07593:493712 339.08453:6684 417.15472:21170 | C21H22O10 | Prunin |
| 4.841 | 245.11743 | -0.55 | 51.10849:5555 54.13312:5463 56.62964:5304 57.05459:5898 57.05736:56703 58.06517:335059 59.06872:7644 59.07270:10761 59.13165:5712 63.54985:5607 71.07298:30200 72.08080:377361 73.08374:12829 85.07571:6145 86.08347:13460 99.09168:174953 100.21899:6042 113.10684:6562 114.11517:132686 119.99220:6388 182.49956:5149 216.83118:5647 226.39394:11642 230.59891:17016 231.10188:156154 231.60323:36435 245.11757:3310519 245.61902:886392 246.11824:16910 | C23H32N6O4S | Vardenafil |
| 4.911 | 725.22736 | -1.9 | 57.03346:154778 58.25559:82756 59.23413:75588 69.03359:223382 71.04912:907065 73.02885:105756 75.04442:106522 81.03350:201993 83.04923:735851 85.02840:3000215 86.03239:77422 97.02819:521835 99.04409:324839 101.05939:91450 103.03900:1428453 105.60831:84092 106.57233:83794 109.02846:668320 111.04390:1008809 123.04387:109665 127.03896:3600462 129.05472:996083 145.04964:1895540 147.04468:194833 147.06564:311141 151.03854:101406 153.01823:1085243 153.05501:92319 163.06052:299902 169.04990:263187 171.02969:121738 187.06012:338358 195.02902:922561 195.06523:95950 219.02933:183042 226.38113:105996 229.07108:164608 237.04700:83262 237.07625:373251 253.07048:137477 255.08762:275128 261.03937:399235 271.08185:305486 273.07571:6269637 285.07565:159615 297.07614:307654 303.05084:93493 305.06525:91508 315.08359:607160 339.08643:538362 348.45923:81553 351.08417:81148 363.08939:221690 371.07629:95338 381.09714:514599 399.11737:124170 405.08633:105309 407.10565:96989 | C33H40O18 | Melitidin |
| 4.924 | 261.11182 | -1.21 | 57.06980:7951 58.06520:34526 59.07291:16240 85.06495:9195 88.03203:5256 91.05433:7465 94.10899:5039 97.62767:5636 117.06940:8573 131.05013:13248 131.08507:5678 148.51070:5562 159.04443:10268 173.06035:8115 175.03984:17805 176.14439:14897 177.05453:39496 187.03865:41209 189.05463:1809629 190.05815:23069 201.05434:33114 203.06961:7409 217.08595:14411 219.06468:6573 226.39430:10383 231.10168:9168 243.10161:310013 261.11075:114987 | C15H18O5 | Meranzin hydrate |
| 5.197 | 305.10165 | -1.03 | 53.53354:4589 58.06522:35194 59.04915:12505 59.07298:20156 60.30303:6338 69.25027:4829 76.63210:5250 79.34138:5092 84.64713:4966 85.06486:9047 91.05444:5640 97.90740:4610 105.03365:6573 106.63098:5138 117.07025:12227 118.04159:6894 176.14328:20143 193.29979:4991 196.28790:6155 203.03412:510904 204.03708:4997 305.10220:588249 305.15143:26796 | C16H16O6 | Heraclenol |
| 5.258 | 475.2117 | -1.05 | 58.06524:43106 59.07307:17819 84.25500:5067 85.02837:5815 94.16692:5155 95.08561:9545 105.06991:10709 109.10252:5390 113.05975:6256 116.64729:5216 117.06985:17422 123.08060:10249 135.11697:22620 136.44740:5580 145.10300:6089 147.11722:23935 149.09622:14400 161.13223:24212 163.11197:15037 176.14305:26719 189.12767:74251 192.13898:8598 199.07904:5485 207.13808:69587 226.38118:11711 226.40594:5159 312.04492:6297 321.86328:5812 390.75189:6038 423.24597:6283 | C22H30N6O4S | Sildenafil |
| 5.613 | 273.07538 | -1.35 | 54.38883:5514 58.06506:23165 59.07284:13632 59.75147:4465 59.93870:6103 69.08075:5123 72.56633:5315 106.89037:4561 117.06998:9404 133.10135:8841 147.04410:114372 153.00273:9860 153.01833:161881 176.14354:9792 179.03416:8421 194.61981:4827 273.07587:853590 273.16632:92435 274.07986:11009 | C15H12O5 | Naringenin |
| 5.654 | 261.11191 | -0.86 | 53.85192:7009 55.94137:7147 57.06982:16113 58.06514:13943 59.07293:10347 69.06996:13644 85.06490:18998 109.74346:7518 110.76395:6391 131.04996:19487 159.04451:20306 161.06010:7718 161.95470:5956 163.03864:6382 173.05937:27457 175.03879:49196 176.14389:12670 177.05455:118173 184.87003:6969 187.03891:128185 187.66220:6692 189.05457:4641767 190.05814:14242 192.13828:9644 201.05478:140945 201.09048:46878 203.07071:18352 205.04970:12870 211.07642:8672 212.08345:8363 215.07065:10388 215.10780:10625 217.08641:27061 219.06374:11244 219.10161:58349 225.51593:7279 228.08012:9036 231.10185:22726 243.10164:1002835 261.11017:389737 | C15H16O4 | Auraptenol |
| 5.834 | 333.16919 | -1.38 | 58.06524:14874 59.07314:8005 69.06993:15699 71.04906:28571 71.92784:5175 77.07732:4561 77.08432:5078 79.05444:6111 81.06995:21405 83.08514:8055 93.06976:51887 95.08574:10553 102.80901:4566 107.08568:49478 109.10138:10703 135.02740:5244 135.11684:189548 136.95164:5172 153.12747:173179 153.14249:9315 163.03908:338754 175.03946:11519 176.14407:7832 187.03789:6168 189.05624:9702 199.03992:11317 201.05426:9183 213.05481:9503 241.08621:5871 255.10223:4989 271.58435:5144 297.14993:6324 333.20291:7025 | C19H24O5 | Marmin |
| 6.011 | 390.14478 | -0.13 | 51.68906:5092 58.06506:5271 69.98487:5336 71.15402:5453 86.49848:4919 96.12589:4882 97.49479:5217 130.06543:20966 135.04410:291319 142.45537:5441 169.07773:16150 197.06950:5364 233.08424:13226 240.11299:60025 247.07080:6128 250.08672:40035 258.80197:5755 262.08658:92801 263.09464:65606 264.10199:46000 268.10825:646810 269.11182:15606 274.08875:5988 302.08160:74456 390.14520:77239 | C22H19N3O4 | Tadalafil |
| 6.102 | 261.11185 | -1.09 | 57.07014:44392 67.05462:38900 69.07020:28715 78.36755:16511 85.06494:83776 89.03487:19721 97.09245:16899 104.23170:19636 124.61196:17137 131.05051:66484 159.04462:75293 161.06046:19352 173.06044:75718 175.03909:145435 177.05473:448039 187.03920:488614 187.07381:40562 189.05473:20125404 190.05797:68947 191.07005:38765 201.05516:421948 203.07045:49560 205.05005:47615 211.07666:30201 212.08237:27812 215.06982:32605 215.10771:29222 217.08632:168991 219.06555:51016 230.07999:27589 231.09987:104734 243.10182:3772952 244.10573:34060 261.11148:1546769 | C15H16O4 | Meranzin |
| 6.567 | 287.2005 | -0.20 | 51.99514:4892 52.93843:5058 58.06506:14084 59.07298:10040 61.67035:5261 63.43159:4809 65.75538:4830 81.30118:5189 81.33423:5306 83.04910:18574 97.06474:340641 107.14960:5478 109.06474:150362 115.33017:4787 123.08050:7261 125.94308:4842 145.10095:7188 159.11574:6349 173.13234:6602 176.14374:6173 185.13225:7177 211.14867:15949 220.98358:5537 226.39355:6298 229.15955:6282 235.41255:4754 251.17906:12374 269.19012:39669 287.20056:987261 287.68164:4980 288.20383:16018 | C19H26O2 | 4-Androstene-3,17-dione |
| 8.073 | 427.28415 | -0.32 | 51.65738:5314 69.07014:5265 88.47796:4632 97.06489:18514 109.06517:5485 109.10110:7363 183.13789:8635 295.20544:7824 313.21564:12741 409.27304:35440 427.28445:102237 | C27H38O4 | Spirost-4-ene-3,12-dion |
| 8.417 | 345.24237 | -0.15 | 53.07654:59247 57.03344:479907 83.04923:160695 85.15096:65288 89.28108:67292 93.06968:103048 95.08573:176369 97.06478:3578705 109.06476:2042512 109.07426:112984 121.10152:140187 123.08028:147135 135.11751:89029 145.10179:127315 147.11705:138112 149.13347:95908 157.10077:127602 159.11728:95769 161.13242:160439 163.14880:133659 171.11679:144733 173.13286:144605 175.14815:558551 177.12738:112648 185.13306:75655 187.14825:145023 189.16377:237712 211.14920:103376 213.16193:110058 252.28276:74888 253.19539:1268882 271.20554:1750503 289.21683:633734 345.24268:4164961 348.52759:86922 | C22H32O3 | Testosterone propionate |

Table S4. Parameters of the PCA and OPLS-DA models

| **Model** | **No. of**  **Components** | **R^2^X** | **R^2^Y** | **Q^2^** | ***p***  **(CV-ANOVA)** | **F**  **(CV-ANOVA)** |
| --- | --- | --- | --- | --- | --- | --- |
| M1, PCA | 8 | 0.91 | - | 0.429 | - | - |
| M2, PCA | 7 | 0.869 | - | 0.455 | - | - |
| M3, PCA | 6 | 0.908 | - | 0.686 | - | - |
| M4, PCA | 6 | 0.738 | - | 0.208 | - | - |
| M5, PCA | 4 | 0.620 | - | 0.373 | - | - |
| M6, OPLS-DA | 1 + 4 | 0.643 | 0.992 | 0.937 | 5 × 10^−23^ | 65 |
| M7, PCA | 7 | 0.848 | - | 0.374 | - | - |


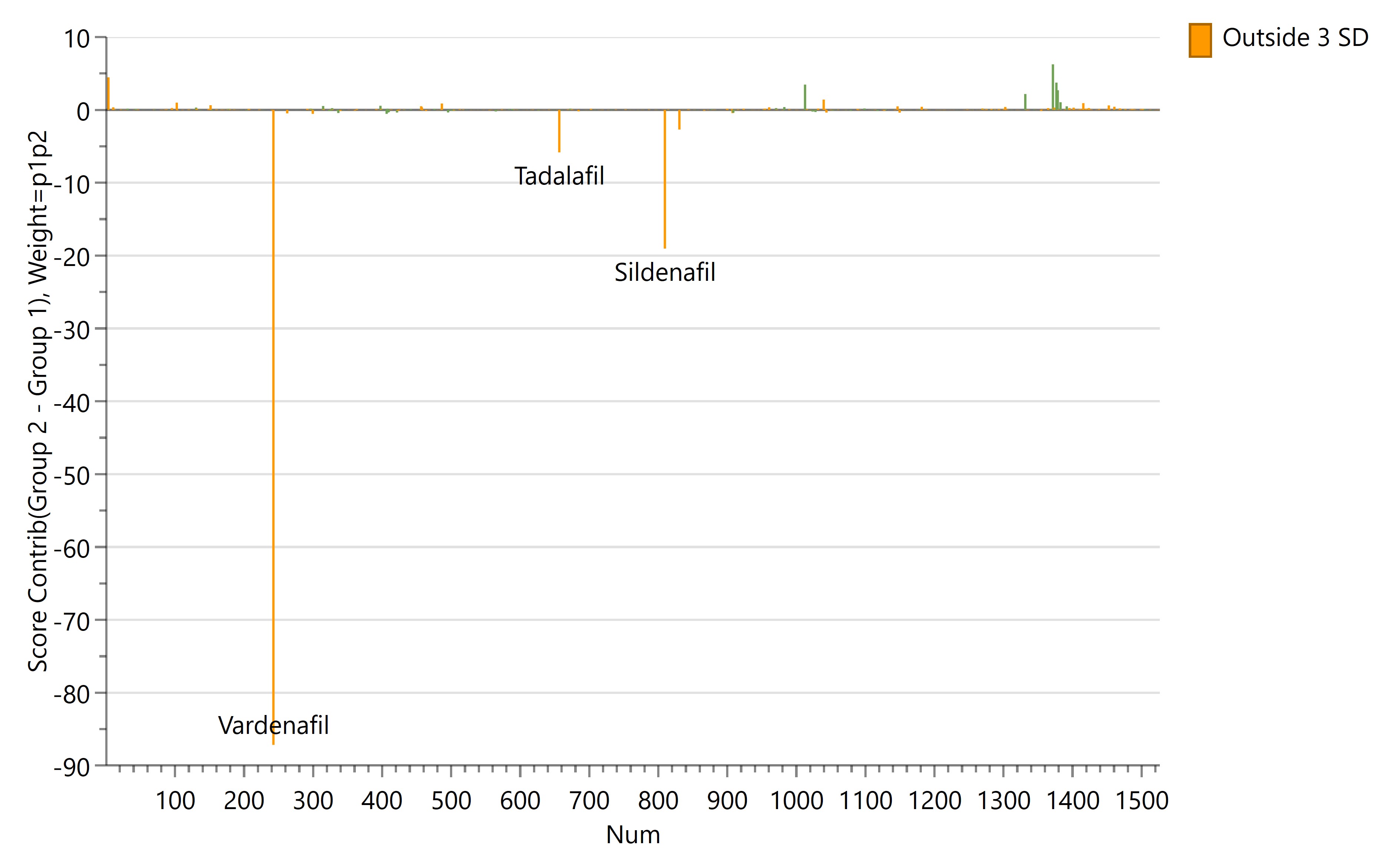


Figure S1. Contribution plot of PCA model M1, identifying the variables that contribute most to the differences observed in two group of samples


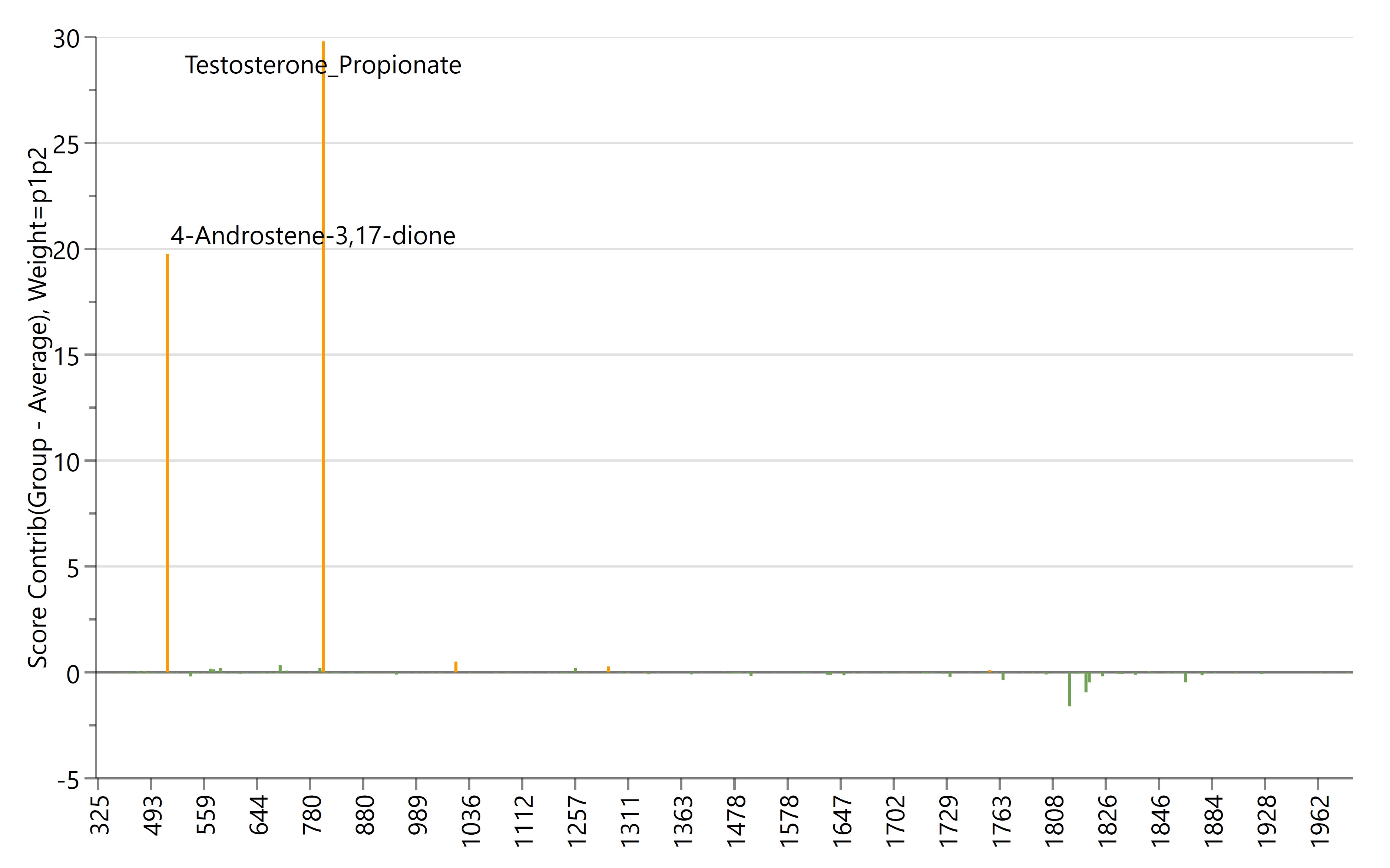


Figure S2. Contribution plot of PCA model M3, identifying the variables that contribute most to the differences observed in two group of samples


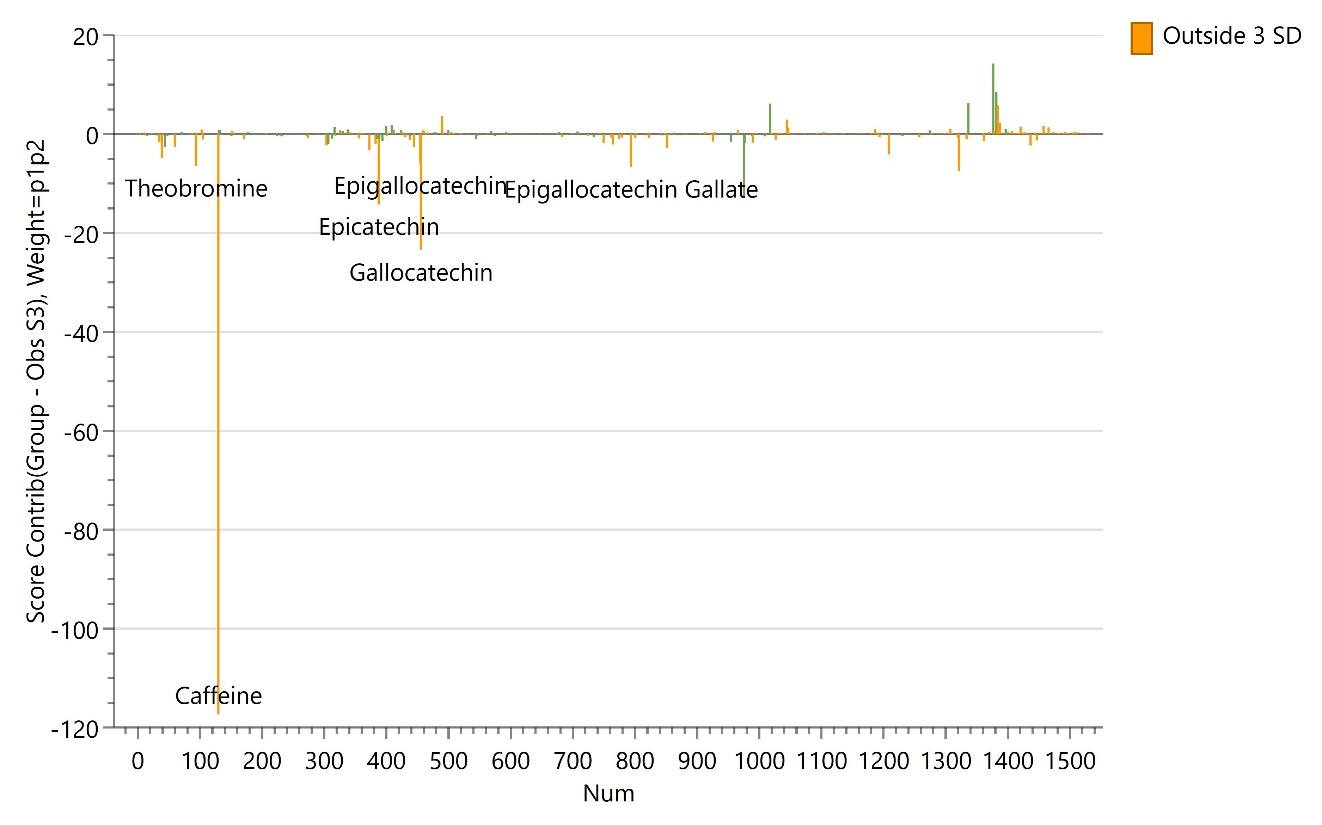


Figure S3. Contribution plot of PCA model M4, identifying the variables that contribute most to the differences observed in outlier S3


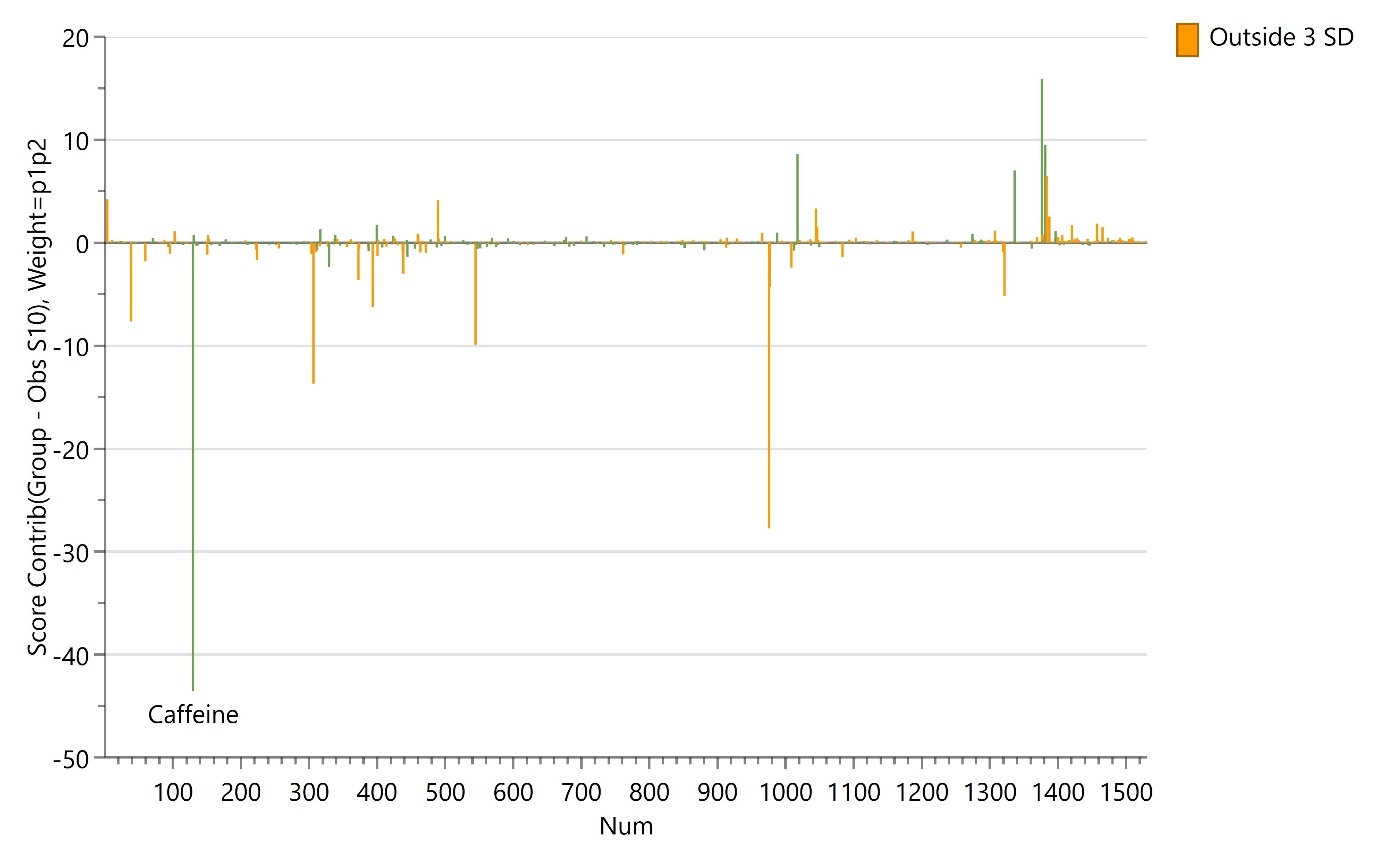


Figure S4. Contribution plot of PCA model M4, identifying the variables that contribute most to the differences observed in outlier S10


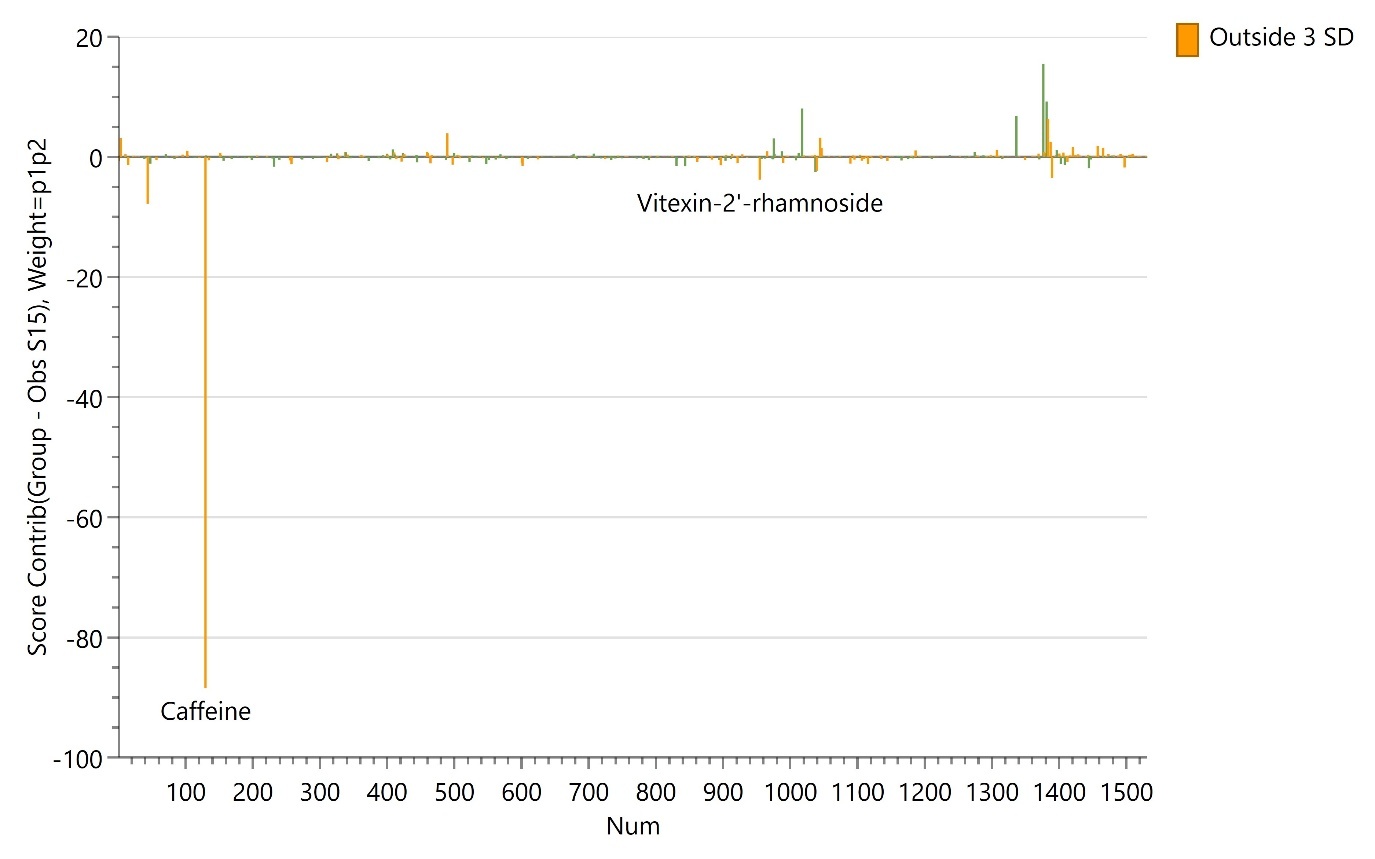


Figure S5. Contribution plot of PCA model M4, identifying the variables that contribute most to the differences observed in outlier S15


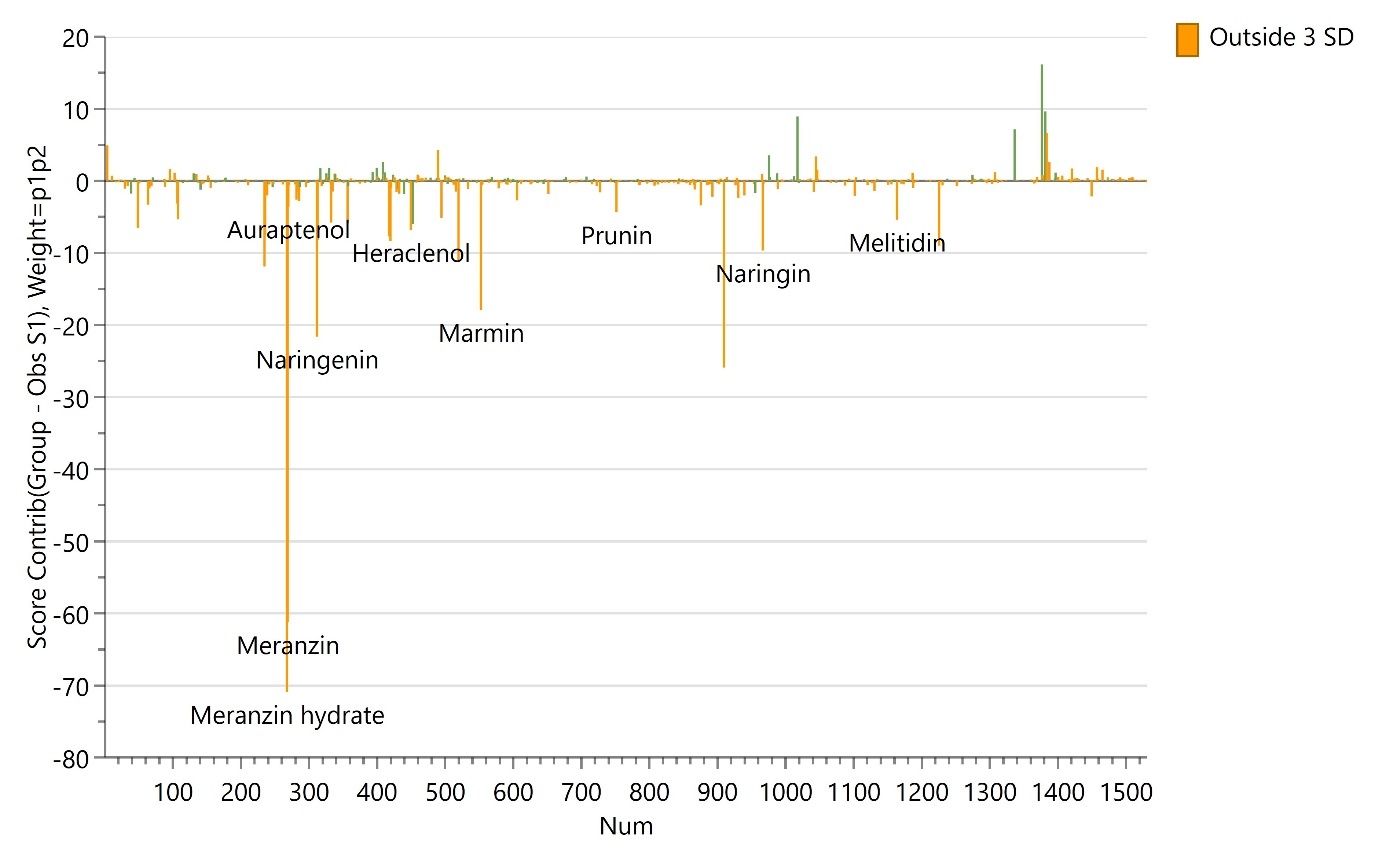


Figure S6. Contribution plot of PCA model M4, identifying the variables that contribute most to the differences observed in outlier S1


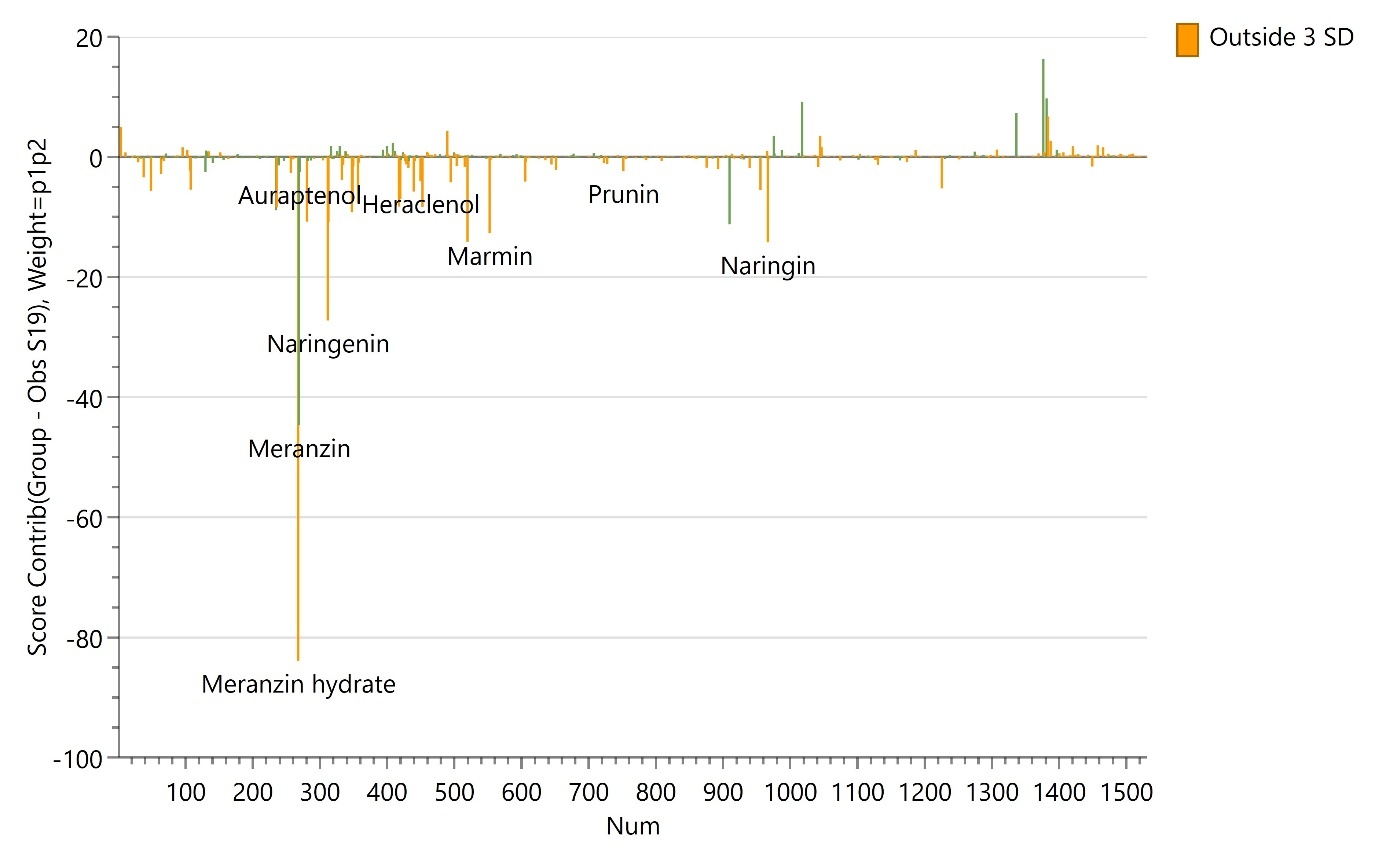


Figure S7. Contribution plot of PCA model M4, identifying the variables that contribute most to the differences observed in outlier S19


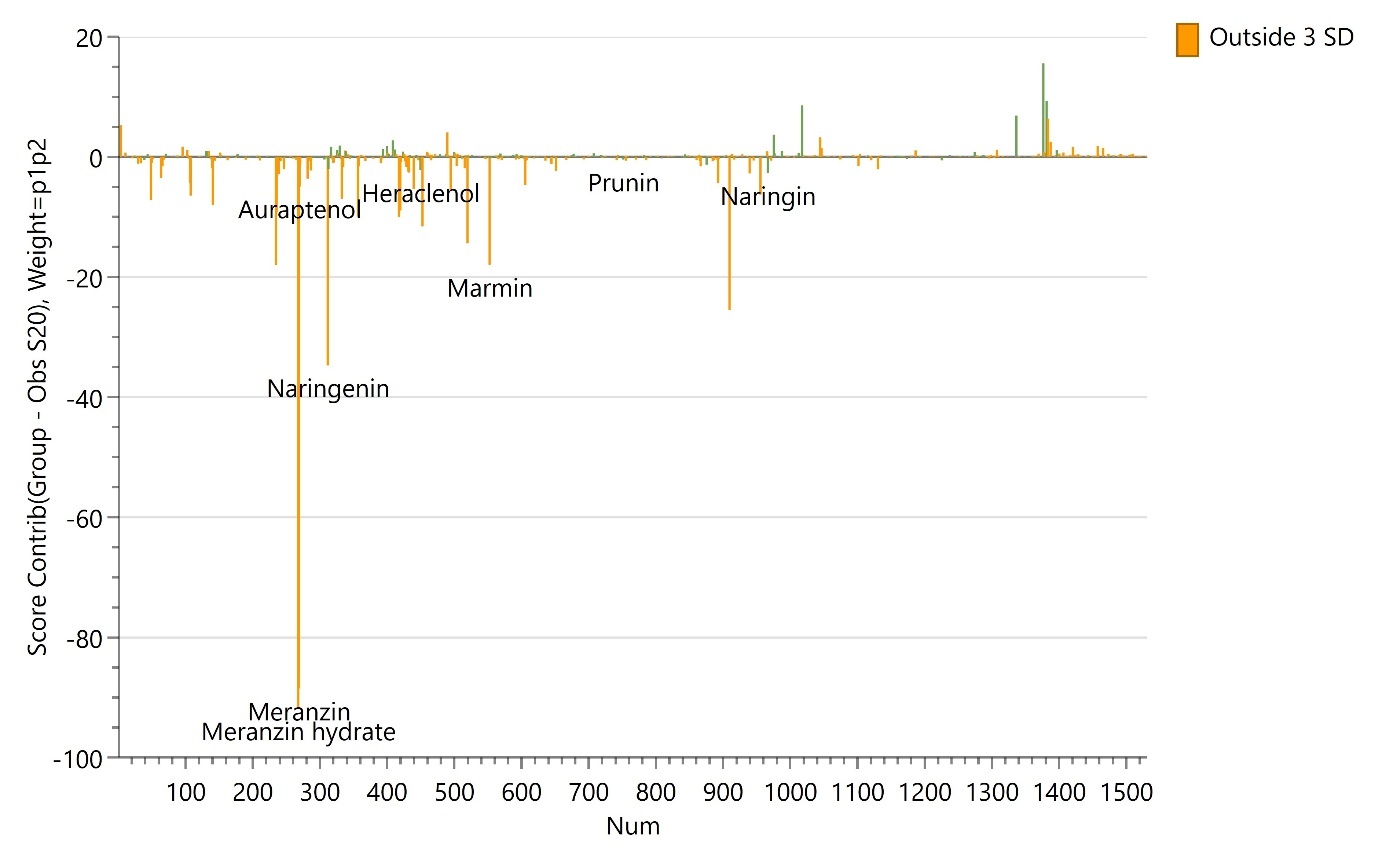


Figure S8. Contribution plot of PCA model M4, identifying the variables that contribute most to the differences observed in outlier S20


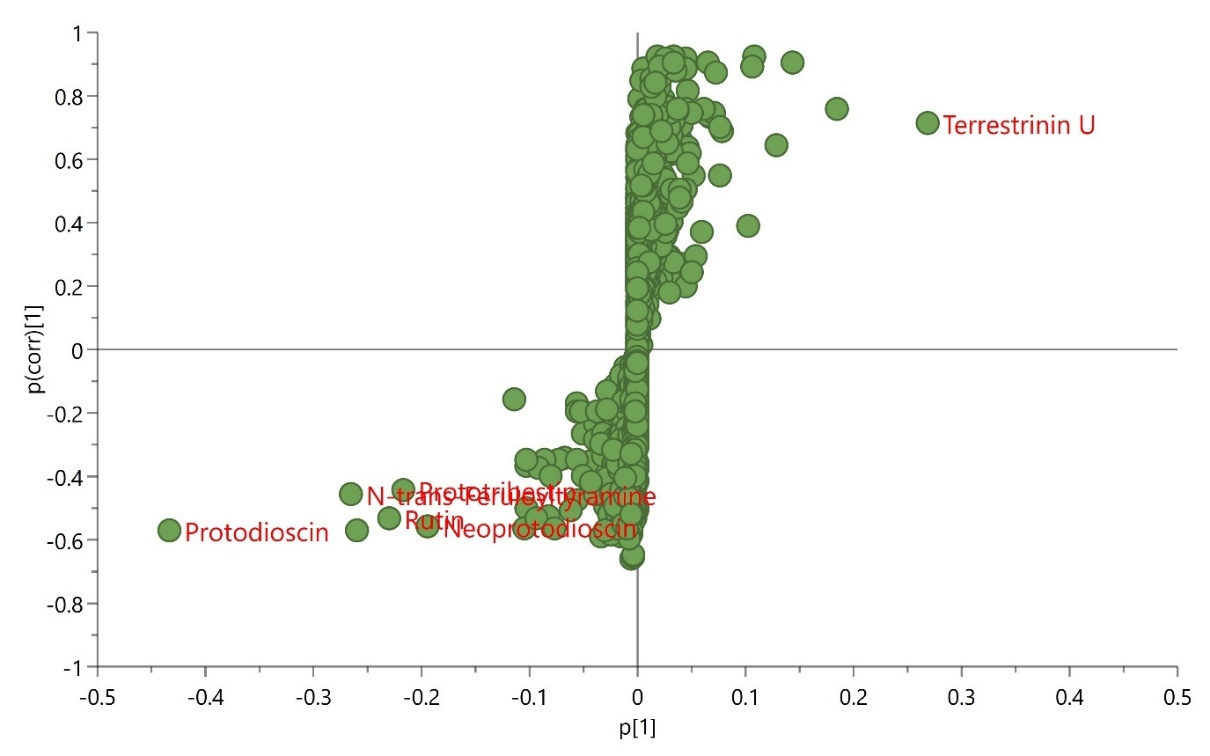


Figure S9. S-plot of OPLS-DA model M6 explaining the variables that contribute most to the differences observed between groups.

Figure S10. Contribution plot of PCA model M7, identifying the variables that contribute most to the differences observed S20 compared to central cluster


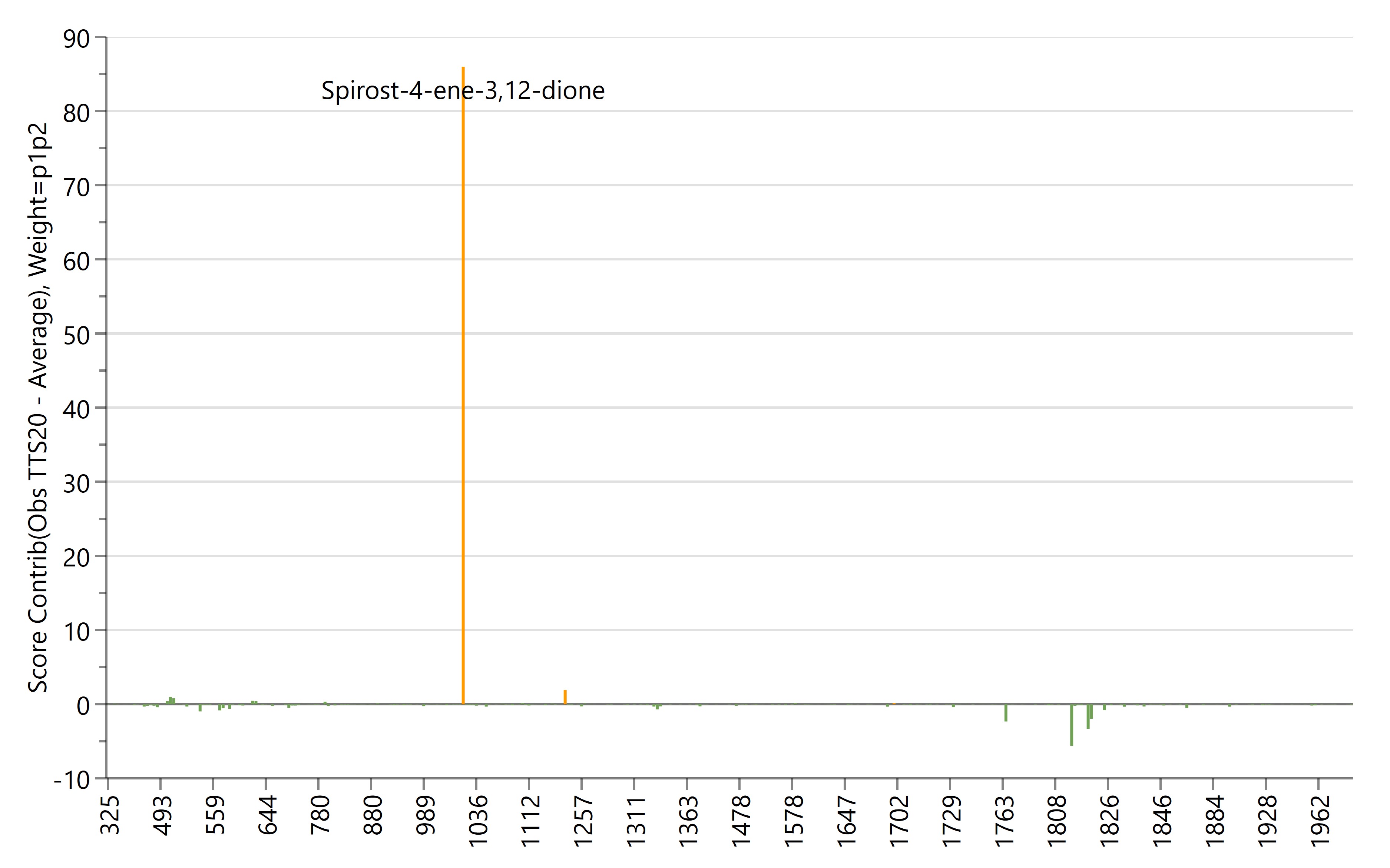


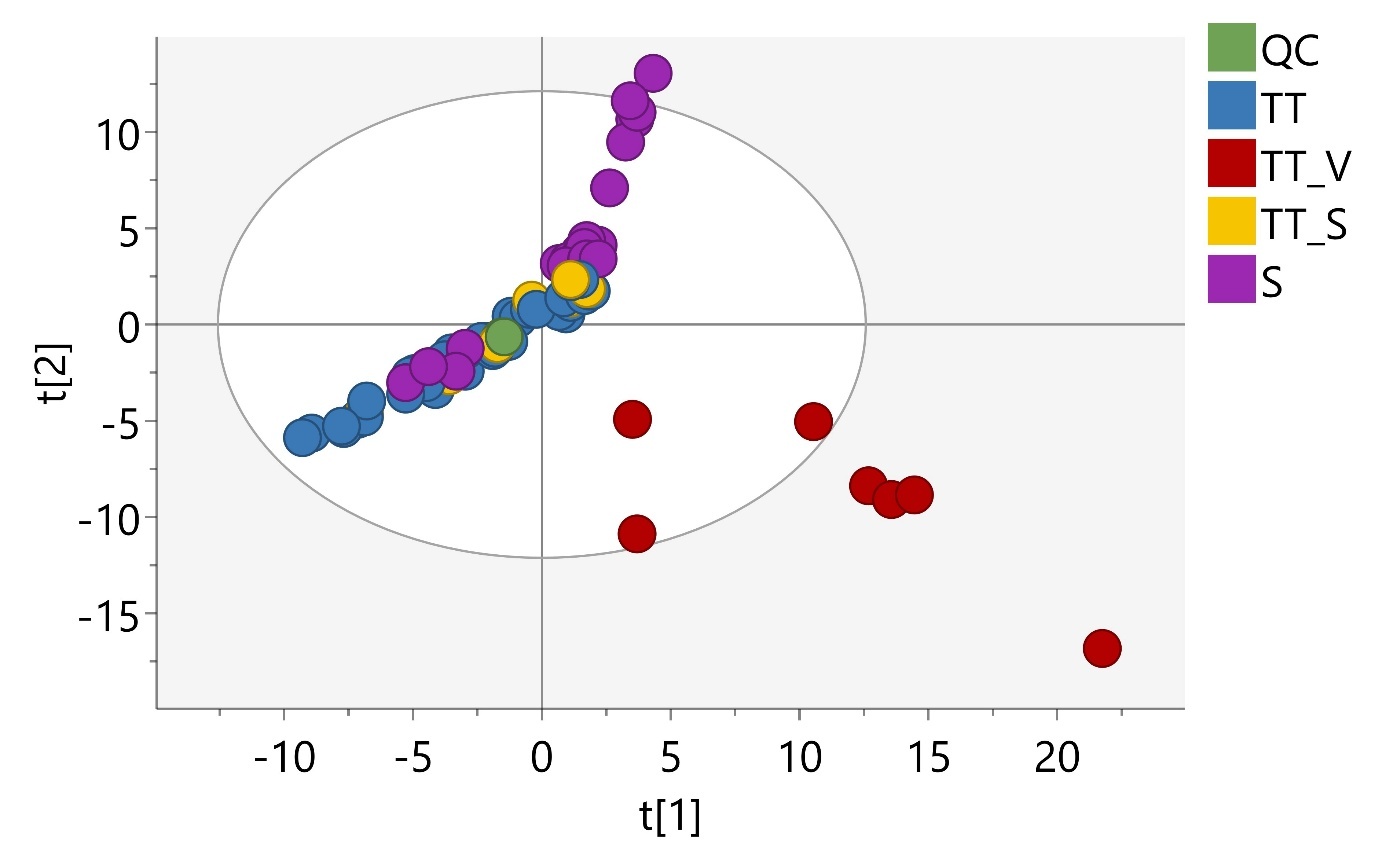


Figure S11. Score plot of PCA model containing: QC samples containing equal amounts of each tested sample, TT-Authentic samples of *T. terrestris*, TT_V- Authentic samples of *T. terrestris* spiked with “PDE5i mix”, TT_S - Authentic samples of *T. terrestris* spiked with “Steroid mix” S- commercially obtained products. The QC samples cluster tightly near the origin, indicating minimal technical variation and reliable data acquisition across analytical runs.

Text S1: Further analysis of supplements performed without prior clean-up with SPE cartridge

The supplements S1, S19, and S20, that have been adulterated with citrus-derived components, along with a negative control (S4), a randomly selected supplement with no suspicions on adulteration, and a positive control CITRAX—a supplement explicitly declared to contain bitter orange (*Citrus aurantium*) extract and 1.25% of synephrine.

Sample preparation

Each sample, 200 mg, was extracted with 1800 µl of a MeOH/H2O (1:1) mixture. The mixtures were sonicated for 10 min in an ultrasonic bath (Elmasonic P 30 H, Germany). After centrifugation at 13,400 rpm for 10 min (MiniSpin Eppendorf, Germany), the supernatants were collected. The final extracts were filtered through a 0.45 µm nylon filter (Agilent, US) before LC-HRMS analysis. The LC-HRMS analysis was conducted as described in the main text in chapter 2.4. LC-HRMS analysis.

Table S5. LC-MS data Further analysis of supplements performed without prior clean-up with SPE cartridge

| **rt** | **name** | **formula** | **mz (MS1)** | **ppm** | **MS2** | **CITRAX, peak area, (estimated content)** | **S1,**  **peak area, (estimated content)** | **S4, peak area, (estimated content)** | **S19, peak area, (estimated content)** | **S20, peak area, (estimated content)** |
| --- | --- | --- | --- | --- | --- | --- | --- | --- | --- | --- |
| 0.54 | **Synephrine** | C9H14NO2 | 168.10158 | 1.91 | **150.09114** (100) | 3.E+09  (≈1.25%)* | 2.E+07  (≈90 ppm)* | not detected | 2.E+06  (≈7ppm)* | 2.E+06  (≈7ppm)* |
| 0.94 | **N-methyltyramine** | C9H14NO | 152.10698 | 0.06 | **121.06466** (100), 152.10692 (23) | 7.E+09 | 5.E+07 | not detected | 7.E+06 | 2.E+07 |

* This value is based on the product declaration where the synephrine content in CITRAX is declared to be 1.25%. The synephrine contents in other products were estimated based on this value and the area under the peak of the MS1 spectrum.
